# Genetic association analysis of the RTK/ERK pathway with aggressive prostate cancer highlights the potential role of *CCND2* in disease progression

**DOI:** 10.1038/s41598-017-04731-4

**Published:** 2017-07-03

**Authors:** Yang Chen, Qin Zhang, Qiuyan Wang, Jie Li, Csilla Sipeky, Jihan Xia, Ping Gao, Yanling Hu, Haiying Zhang, Xiaobo Yang, Haitao Chen, Yonghua Jiang, Yuehong Yang, Ziting Yao, Yinchun Chen, Yong Gao, Aihua Tan, Ming Liao, Johanna Schleutker, Jianfeng Xu, Yinghao Sun, Gong-Hong Wei, Zengnan Mo

**Affiliations:** 1 Center for Genomic and Personalized Medicine, Guangxi Medical University, Nanning, Guangxi Zhuang Autonomous Region China; 2 Department of Urology and Nephrology, the First Affiliated Hospital of Guangxi Medical University, Nanning, China; 3 Guangxi key laboratory for genomic and personalized medicine, Guangxi collaborative innovation center for genomic and personalized medicine, Nanning, Guangxi Zhuang Autonomous Region China; 4 Biocenter Oulu, University of Oulu, Oulu, Finland; 5 Faculty of Biochemistry and Molecular Medicine, University of Oulu, Oulu, Finland; 6 The Guangxi Zhuang Autonomous Region Family Planning Research Center, Nanning, Guangxi China; 7 Department of Medical Biochemistry and Genetics, University of Turku, Turku, Finland; 8 Medical Research Center, Guangxi Medical University, Nanning, Guangxi China; 9 Program for Personalized Cancer Care, NorthShore University HealthSystem, Evanston, Illinois USA; 10 Tyks Microbiology and Genetics, Department of Medical Genetics, Turku University Hospital, Turku, Finland; 11 Fudan Institute of Urology, Huashan Hospital, Fudan University, Shanghai, China; 12 Department of Urology, Shanghai Changhai Hospital, The Second Military Medical University, Shanghai, China

## Abstract

The RTK/ERK signaling pathway has been implicated in prostate cancer progression. However, the genetic relevance of this pathway to aggressive prostate cancer at the SNP level remains undefined. Here we performed a SNP and gene-based association analysis of the RTK/ERK pathway with aggressive prostate cancer in a cohort comprising 956 aggressive and 347 non-aggressive cases. We identified several loci including rs3217869/*CCND2* within the pathway shown to be significantly associated with aggressive prostate cancer. Our functional analysis revealed a statistically significant relationship between rs3217869 risk genotype and decreased *CCND2* expression levels in a collection of 119 prostate cancer patient samples. Reduced expression of *CCND2* promoted cell proliferation and its overexpression inhibited cell growth of prostate cancer. Strikingly, *CCND2* downregulation was consistently observed in the advanced prostate cancer in 18 available clinical data sets with a total amount of 1,095 prostate samples. Furthermore, the lower expression levels of *CCND2* markedly correlated with prostate tumor progression to high Gleason score and elevated PSA levels, and served as an independent predictor of biochemical relapse and overall survival in a large cohort of prostate cancer patients. Together, we have identified an association of genetic variants and genes in the RTK/ERK pathway with prostate cancer aggressiveness, and highlighted the potential importance of *CCND2* in prostate cancer susceptibility and tumor progression to metastasis.

## Introduction

Prostate cancer remains the second most commonly diagnosed cancer and one of the leading causes of cancer-related deaths in men worldwide. More than 1.1 million cases of prostate cancer and 307,000 deaths were recorded around the globe in 2012, accounting for approximately 15% of new cancer cases in men and 6.6% of the total male cancer mortality^1^, respectively. In the United States, there are nearly 180,890 new cases diagnosed and around 26,120 deaths in 2016^2^. While prostate cancer patients with an indolent form of the disease may be easily treated, the patients who have aggressive prostate cancer indicate worse prognosis and thus need intensive treatment. Recent genome-wide association studies (GWASs) have been applied to identify possible prostate cancer risk loci with potential in management of the disease, though the molecular pathogenesis is being unraveled^3–6^, especially for the aggressive form of the disease.

Many established risk factors have been implicated in prostate cancer etiology and development. These include genetics and family history, ethnic background, lifestyle, advancing age and environment. In addition, considerable evidence indicated that the signaling pathways including AR, PI3K/AKT, and RTK/ERK^7–9^ have been identified as the key factors for prostate cancer risk, yet the underlying genetic basis remains elusive. The association analyses in our previous work suggested that the RTK/ERK pathway might be a significant factor conferring risk of prostate cancer^10–12^. Moreover, recent studies demonstrated the important role of this pathway in metastasis and progression of prostate cancer^13, 14^. However, the potential genetic risk loci, genes and their function in this pathway associated with aggressive prostate cancer has not been defined.

With a number of genes^15^ such as *EGFR*, *PDGF*, *GRB2*, *MET* and cyclin D genes^16^ (*CCND1*, *CCND2* and *CCND3*) involved, the RTK/ERK pathway regulates various biological processes including cellular signal transduction and crosstalk, cell proliferation, differentiation, survival and migration in normal and cancer development^17, 18^. Mutations that induce abnormalities of the structures of proteins in the pathway, may lead to cell transformation and malignancy in various types of cancer. The receptor tyrosine kinases (RTKs) lie upstream in the pathway, transduce the signal into the cells and trigger a cascade of phosphorylation for the downstream kinases such as the mitogen-activated protein kinase (MAPK) cascade, including Raf, MEK and extracellular signal-regulated kinase (ERK) kinases^17, 18^. The consecutive signal transduction may lead to the phosphorylation of target proteins inside the cell and control normal biological activities. In contrast, the imbalance during the cascade of signal transduction and the inappropriately response for susceptible environment stimulus may lead to various diseases, including cancer. Therefore, investigation on the associations of the RTK/ERK pathway with prostate cancer risk can provide new insight into the molecular mechanisms underlying the susceptibility, and development of prostate cancer at the pathway-level, and reveal novel links between the pathway genes and disease progression to advanced stage and metastasis.

Although the potential association of the RTK/ERK pathway with prostate cancer risk has been suggested by our previous study^10–12^, the genetic and functional link of the pathway to aggressive prostate cancer have not been elucidated. In this work, on the basis of the Chinese Consortium for Prostate Cancer Genetics (ChinaPCa) project, we performed SNP- and gene-based prostate cancer case-only study, as well as expression quantitative trait loci (eQTL), clinical correlation, and prognosis analyses. Whereas some loci and genes were defined, only one SNP (rs3217869 in *CCND2*) was reproducibly identified to be significantly associated with aggressive prostate cancer by multiple testing. The eQTL analysis revealed a significant association of the risk allele at rs3217869 with decreased mRNA levels of *CCND2*. Interestingly, we found that *CCND2* is strikingly downregulated in advanced prostate tumors and its downregulation is associated with disease progression and poor prognosis in multiple independent cohorts of prostate cancer, suggesting that *CCND2* is a new biomarker to distinguish aggressive disease and a potential metastatic tumor suppressor. Thus, our study not only investigated the effects of the RTK/ERK pathway on the progression of prostate cancer, but also revealed the genetic factors responsible for aggressive disease susceptibility, highlighted the potential role of *CCND2* in disease progression.

## Materials and Methods

### Patients and study approval

Informed consent was obtained from all subjects. The experimental protocols for the study using the China PCa cohort was approved by the institutional review boards at Shanghai Changhai Hospital, The Second Military Medical University, Shanghai, China. The study using Finnish PCa cohort was performed with the appropriate research permissions from the Ethics Committee of the Tampere University Hospital, Finland, and the Ministry of Social Affairs and Health in Finland. All experiments and methods were performed according to these relevant guidelines and regulations.

### Study population

The Chinese Consortium for Prostate Cancer Genetics (ChinaPCa) has recruited a study population comprised of Han Chinese men from southeastern China, with various areas including Shanghai, Suzhou, Guangxi and Nanjing. In current study, 1417 prostate cancer patients from Shanghai and surrounding areas genotyped in the first state of a previous GWAS^19^ were collected with the information of age, PSA level, Gleason score (GS) and clinical stage. The details of this cohort were presented in the study by Xu *et al*.^19^. To ensure that all of the SNPs in the RTK/ERK signaling pathway genes were covered, the region around each gene was extended by 100 kb. Finally, 50,459 single nucleotide polymorphisms (SNPs) were identified.

To investigate the association between the RTK/ERK pathway and aggressive prostate cancer, this analysis was designed to be a case-only study, in which the samples were divided into two groups: aggressive cases and non-aggressive prostate cancer cases. The aggressive cases were defined as: histologically confirmed prostate cancer, with GS ≥ 8, or clinical stage ≥T3, or with lymphatic metastasis (N+), or being metastatic (M+), or with PSA >50 ng/ml. Once the unambiguous samples met any of the above criteria, the aggressive data will be collected. Otherwise, the non-aggressive prostate cancer was defined. Finally, after the information filtering, the study included 1303 prostate cancer patients consisting of 956 aggressive and 347 non-aggressive ones^20, 21^. The characteristics of the collected samples are presented in Table 1.

**Table 1:** The characteristics of the samples we included in our analysis.

	aggressive (n = 956)	non-aggressive (n = 347)	P
^a^Age (mean ± SD, n)	71.25 ± 8.38, 950	70.76 ± 7.37, 343	0.309
^b^PSA (mean ± SD, n),	152.79 ± 517.55, 855	15.09 ± 9.48, 347	<0.001
Classification of PSA, n (%)			
≤4.0	31 (3.24%)	18 (5.19%)	
>4.0, <10	69 (7.22%)	105 (30.26%)	
≥10	833 (87.13%)	224 (64.55%)	^d^<0.001
Undefined	23 (2.41%)	0 (0.00%)	^e^<0.001
^c^Gleason score, n (%)			
≤7	396 (41.42%)	347 (100%)	
≥8	537 (56.17%)	0 (0.00%)	^f^<0.001
Undefined	23 (2.41%)	0 (0.00%)	^g^<0.001
Clinical stage, n (%)			
Stage (I, II)	353 (36.92%)	347 (100%)	
Stage (III, IV)	491 (51.36%)	0 (0.00%)	^h^<0.001
Undefined	112 (11.72%)	0 (0.00%)	^i^<0.001

^a^The information of age were missing in 6 aggressive and 4 no-aggressive samples.

^b^The levels of PSA in 78 aggressive samples were too high and no measurable values; In addition, 23 aggressive samples were missing.

^c^23 aggressive samples were missing for Gleason score.

^d^The Χ2-test for three levels of PSA (PSA ≤ 4.0, 4 < PSA < 10, PSA ≥ 10) in aggressive and non-aggressive groups.

^e^The Χ2-test for thre^e^ levels of PSA (PSA ≤ 4.0, 4 < PSA < 10, PSA ≥ 10 and Undefined) in aggressive and non-aggressive groups.

^f^The Χ2-test for three levels of Gleason score (GS ≤ 7, GS ≥ 8) in aggressive and non-aggressive groups.

^g^The Χ2-test for three levels of Gleason score (GS ≤ 7, GS ≥ 8 and Undefined) in aggressive and non-aggressive groups.

^h^The Χ2-test for three levels of Clinical stage (Stage (I, II) and Stage (III, IV)) in aggressive and non-aggressive groups.

^i^The Χ2-test for three levels of Clinical stage (Stage (I, II) and Stage (III, IV) and Undefined) in aggressive and non-aggressive groups.

### SNP selection and association analysis

On the basis of ChinaPCa, the genotype data for the 40 genes in the RTK/ERK pathway such as *EGFR*, *EGF*, *HGF*, *HRAS* and cyclin D genes (*CCND1*, *CCND2* and *CCND3*) were collected. Before conducting the association analysis, tag SNPs representing each loci in the RTK/ERK pathway relevant genes were selected using the web server SNPinfo^22^ (http://manticore.niehs.nih.gov/snpinfo/snptag.htm), which includes a set of web-based SNP selection tools with many functional modules involved, such as GWAS SNP Selection in Linkage Loci, Candidate Gene SNP Selection, TAG SNP Selection, SNP Function Prediction and so on^22^. On the basis of the powerful system, the official gene names and IDs were obtained using the HapMap database and CHB + JPT populations. Then, tag SNPs were calculated with common SNPs (minor allele frequency, MAF ≥ 0.05; r^2^ ≥ 0.8). Finally, 760 SNPs representing the pathway relevant genes were collected. In addition, the genotype data of the corresponding tag SNPs were extracted using PLINK tool^23^.

### Statistical analysis

In this study, two groups of samples were defined. One is aggressive prostate cancer and the other is non-aggressive on the basis of the criteria as described above. Then, the information of ages, PSA levels, GS and Clinical stage were collected. The Student’s t test, Mann-Whitney U test, and the Χ^2^-test were properly applied.

To investigate the real association between the SNPs in the RTK/ERK pathway and aggressive prostate cancer, we adopted three genotype models, including dominant, recessive and additive model (http://pngu.mgh.harvard.edu/~purcell/plink/anal.shtml#model)^23^. The odd ratios (OR) and corresponding 95% confidence intervals (CI) were used to evaluate the power of associations. Meanwhile, Cochran–Armitage trend test was also applied (http://pngu.mgh.harvard.edu/~purcell/plink/anal.shtml#model)^23^. In addition, to discover the independent association between the RTK/ERK pathway relevant loci and aggressive prostate cancer, those SNPs in each gene with the lowest *P* values were collected as the covariates in the next conditional analysis (http://pngu.mgh.harvard.edu/~purcell/plink/anal.shtml#glm)^23^. All the associations were conducted by adjusting the parameter of age for every subject. In addition, the associations between the discovered loci and clinical parameters (especially for GS and clinical stage) were also conducted, which would help understand the loci more clearly on the level of possible pathogenesis. However, in our data, some of the PSA levels were a scope without pronounced values, which were only enough to define the aggressive and non-aggressive prostate cancer. Thus, we did not include these in this analysis to rule out potential bias. All the statistical analyses were performed using SPSS version 16.0 software (SPSS Inc., Chicago, IL, USA) and PLINK^23^ program. The statistical tests were two-tailed.

### Gene-based pathway analysis

After testing the association at the SNP-level for aggressive and non-aggressive prostate cancer, the gene-based pathway analysis was conducted for further exploration using the adaptive rank truncated product (ARTP) method^24^. This method could potentially remedy the results discovered by single-SNP analysis and provide additional insights into the genetic mechanism underlying complex diseases. The analysis consisted of two steps: firstly, the standardized summary was acquired for the evidence of association between a gene and the outcome; secondly, these gene-level P-values were combined into a test statistic for the disease-pathway association. To use the method, three sets of information are needed: the genotypes of the SNPs, the phenotype for the samples, and the annotation of the SNPs. According to the manual of ARTP, the calculations were mainly divided into three steps: (1) Defining the phenotype and genotype data; (2) Calling the ARTP function; and (3) Calling the runPermutations and ARTP pathway functions. Then, in this study, the gene-based pathway association was conducted with 10,000-fold permutations to make the results more accurate. In all of the analyses, *P*-values were two-sided with R v2.13.2.

### Association analysis of SNP genotype with gene expression

For the analysis of the association between the rs3217869 genotype and *CCND2* expression levels, Kruskal–Wallis H test was applied to assess the statistical significance. Furthermore, we also used Matrix eQTL to test for the cis eQTL association between rs3217869 genotypes and *CCND2* expression levels using data from Camcap cohort comprised of 119 prostate samples^4, 25^. The parameters “useModel = modelLINEAR”, “errorCovariance = numeric ()” were used with Matrix eQTL. R (version 3.2.2) was used to graphically visualize the association between rs3217869 genotypes and *CCND2* expression levels.

### Cell lines and transfection

The prostate cancer cell lines LNCaP, PC3 and DU145 were grown in Dulbecco’s Nodified Eagle medium containing 10% fetal calf serum (FCS) and 1.2% penicillin/streptomycin (Gibco, Thermo Fisher scientific), at 37 °C and 5% CO2. Considering the expression of CCND2 was lower in LNCaP cells than DU145 and PC3 (data not shown), LNCaP cell line was selected to perform the CCND2 overexpression experiment. The full-length of human CCND2 cDNA were cloned into pcDNA3.1(+) expression vector (Life technologies, Carlsbad, CA) and the construct was verified by sequencing. pcDNA3.1-CCND2 or parental pcDNA3.1 vector were transfected into LNCaP cells using Lipofectamine 2000 (Invitrogen) according to the manufacturer’s protocol.

### Western blot

For detection of CCND2 overexpression in LNCaP cells were collected with a gum rubber-scraping device, lysed with RIPA Lysis Buffer (P0013C, Beyotime Institute of Biotechnology) and protein concentration was determined using BCA assay (Thermo Scientifc, Waltham, MA, USA) according to manufacturer’s information. Subsequently, 50 μg of total protein were separated by SDS-PAGE, transferred to nitrocellulose membranes and incubated with antibodies against anti-CCND2 (dilution: 1:4000, Cell Signaling, Danvers, MA, USA). Proteins levels were normalized using Actin levels as reference.

### siRNA transfection and quantitative RT-PCR

To further evaluate functional role of *CCND2* in prostate cancer cell growth, we performed short interfering RNA (siRNA)-mediated knockdown assays by transfecting siRNAs against *CCND2* into the DU145 cells that were seeded in 96-well palates (reverse transfection, 6pmol siRNA per well) using HiPerFect Transfection Reagent (Cat No: 301705). The siRNAs used included *CCND2* siRNAs: AGGAGTGTAGTTGGATCTCTA (SI00027839); AAGAAATAGACTTGCACCTTA (SI00027853); CAGGGCCGTGCGGGACCGCAA (SI03071369) and AllStars Hs Cell Death siRNA (SI04381048) and AllStars Negative Control siRNA(SI03650318) purchased from Qiagen. We used qRT-PCR (SYBR Select Master Mix, 4472920, Applied Biosystems) to evaluate knockdown effect of the siRNAs on CCND2. Primer sequences used in this experiment are TCCTGGCCTCCAAACTCAAAG (*CCND2* forward), GAGGCTTGATGGAGTTGTCG (*CCND2* reverse) and AGAAAATCTGGCACCACACC (*ACTB* forward) and AGAGGCGTACAGGGATAGCA (*ACTB* reverse).

### Cell proliferation assay

3-(4,5-dimethyl-2-thiazolyl)-2,5-diphenyl-2-H-tetrazolium bromide assay (MP Biomedicals, LLC) was applied to evaluate the cell viability. Transfected LNCaP cells were seeded in 96-well plates. MTT was added to each well, incubating for 4 h at 37 °C. Then, using dimethyl sulfoxide (DMSO, 100 µL), the precipitate was dissolved. The absorbance at 570 nm was measured with EPOCH2 Microplate Readers (BioTek). The measures were performed at 0 h, 24 h and 48 h. All groups were consisted of 3 wells of LNCaP cells per time point independently (four times in total). For siRNA-mediated knockdown cell proliferation experiments, cell viability and proliferation were determined by using XTT (11465015001, Roche) and values were obtained at designed time point by measuring the absorbance at 450 nm. Three independent experiments have been run, and in each experiments, each group include 3 wells of DU145 cells at given time points. Two-tailed t test was used to calculate the significance.

### Differential gene expression analysis

For the differential gene expression analysis, we performed Mann-Whitney U tests or Kruskal–Wallis H test to evaluate the significance for the comparison of *CCND2*, *PDGF-C* and other gene expression levels among normal, tumor or metastasis samples from Oncomine database^26^. R (version 3.2.2) was used to perform these statistical analyses and box plot was used to graphically display the distribution of gene expression among different groups.

### Immunohistochemistry

Aggressive prostate cancer (n = 5), non-aggressive prostate cancer (n = 4), benign prostate hyperplasia (BPH, n = 3) and one normal bladder were collected from the First Affiliated Hospital of Guangxi Medical University, Nanning, China. The study was approved by the ethics committee. All the formalin-fixed and paraffin-embedded prostate tissue sections were performed with DAB Detection Kit (Streptavidin-Biotin) according to the instructions. The anti-CCND2 (Cell Signaling, Danvers, MA, USA) was used at a 1:800 dilution. With Meyer’s hematoxylin, the sections were analyzed with light microscopy.

### Analysis of association between *CCND2* expression and risk of overall survival or biochemical recurrence

We assessed the association between *CCND2* expression levels and prostate cancer overall survival and biochemical recurrence by using Kaplan–Meier estimator as previously reported^27^. For the association analysis of *CCND2* expression levels and overall survival and biochemical recurrence, we used a collection of 596 prostate carcinoma samples from the Nakagawa Prostate dataset from Oncomine database^26, 28^. Samples were stratified into two groups based on median measure of *CCND2* expression levels. For a given sample expression measurement, the expression value was subtracted by the median value of all sample expression measurements. Thus, scores above zero indicate higher expression while scores below zero indicate lower expression. We then ranked samples in each group according to *CCND2* expression and defined the higher *CCND2* expression group of top 50% of samples with positive scores. In a same way, we defined lower *CCND2* expression group of bottom 50% of samples with negative scores.

### Multivariate analysis

For analysis of the association between prostate cancer overall survival, biochemical recurrence and *CCND2* expression and clinical variables including Gleason score (GS), PSA, T stage and age, we performed multivariate Cox proportional hazard analyses with the 596 samples from the Nakagawa prostate dataset from Oncomine database^26, 28^ in R (version 3.2.2). Samples were stratified into two groups with higher and lower expression by comparing to the median measurement of *CCND2* expression. Samples were ranked based on *CCND2* expression and highest 50% and lowest 50% of the samples were selected for the Cox regression analysis. The clinical relevance of overall survival and biochemical recurrence and covariates were performed in several different scenarios. We tested the association by including covariates Gleason score, PSA, age, and clinical T state.

## Results

### Characteristics

Prostate cancer is a common disease, affecting a large number of males worldwide. As an invasive form of the disease, aggressive prostate cancer can induce more serious damage than other forms. In our previous work^11, 12^, the potential link between the prostate cancer risk and the RTK/ERK pathway was observed. On the basis of these associations, we proposed to illustrate the effects of the pathway on aggressive prostate cancer, which could help understand the pathogenesis and the genetic basis underlying the disease susceptibility and progression.

### SNP-based association analysis

As the iconic SNPs in a region of the human genome, the tag-SNPs were calculated with the SNPinfo^22^ for every gene in the RTK/ERK pathway. Finally, we obtained 760 tag SNPs. The association analysis was conducted using three common genotype models and the logistic regression after adjusting for age. For the additive model, 32 loci were discovered with *P* < 0.05, 31 for dominant model and 21 for recessive model (Table 2 and Supplementary Table 1), respectively. In the additive and dominant model, the lowest *P* value was 1.132 × 10^−3^ (OR = 1.393, 95% (confidence interval) CI = 1.141–1.700) and 9.800 × 10^−4^ (OR = 1.519, 95% CI = 1.185–1.948) for rs603781 in the *PDGF-D* gene (Supplementary Table 1). As for the recessive model, rs11247380 in *IGF1R* (*P* = 2.269 × 10^−3^, OR = 1.757 95% CI = 1.224–2.524) was suggested to be the most significant (Supplementary Table 1).

**Table 2:** The association between aggressive prostate cancer and the loci of the RTK/ERK pathway assessed by the additive, dominant and recessive models, respectively.

CHR	Gene	SNP	BP	A1	Model	OR (95% CI)	P^a^	Conditional SNP	Conditional P value^b^	P^e^
4	EGF	rs2255355	110891543	A	Additive	1.186 (0.991–1.421)	6.330 × 10^−2^	—	—	6.528 × 10^−2^
					Dominant	1.095 (0.853–1.406)	4.770 × 10^−1^	—	—	
					Recessive	1.694 (1.147–2.504)	8.125 × 10^−3^	REF^c^	REF	
		rs1860129	110886343	G	Additive	1.136 (0.942–1.369)	1.814 × 10^−1^	—	—	1.787 × 10^−1^
					Dominant	1.020 (0.797–1.305)	8.777 × 10^−1^	—	—	
					Recessive	1.841 (1.164–2.912)	9.107 × 10^−3^	rs2255355	3.404 × 10^−1^	
7	EGFR	rs1050171	55249063	A	Additive	0.707 (0.557–0.897)	4.306 × 10^−3^	REF^c^	REF	4.795 × 10^−3^
					Dominant	0.667 (0.511–0.871)	2.890 × 10^−3^	REF^c^	REF	
					Recessive	0.763 (0.326–1.784)	5.321 × 10^−1^	—	—	
		rs2075101	55250026	G	Additive	0.719 (0.567–0.911)	6.367 × 10^−3^	rs1050171	NA	7.148 × 10^−3^
					Dominant	0.681 (0.523–0.888)	4.572 × 10^−3^	rs1050171	NA	
					Recessive	0.764 (0.326–1.787)	5.342 × 10^−1^	—	—	
11	PDGF-D	rs603781	103965133	T	Additive	1.393 (1.141–1.700)	1.132 × 10^−3^	REF^c^	REF	1.415 × 10^−3^
					Dominant	1.519 (1.185–1.948)	9.800 × 10^−4^	REF^c^	REF	
					Recessive	1.477 (0.919–2.376)	1.075 × 10^−1^	—	—	
12	CCND2	rs3217892	4403864	A	Additive	1.488 (1.139–1.943)	3.542 × 10^−3^	REF^c^	REF	3.438 × 10^−3^
					Dominant	1.548 (1.143–2.097)	4.729 × 10^−3^	REF^c^	REF	
					Recessive	1.970 (0.815–4.763)	1.322 × 10^−1^	—	—	
		**rs3217869**	**4399970**	**A**	**Additive**	**1**.**511 (1**.**135–2**.**010)**	**4**.**653 × 10** ^**−3**^	rs3217892	7.263 × 10^−3^	5.188 × 10^−3^
					**Dominant**	**6**.**907 (1**.**733–26**.**900)**	**5**.**341 × 10** ^**−3**^	rs3217892	8.938 × 10^−3^	
					**Recessive**	**1**.**435 (1**.**055–1**.**954)**	**2**.**156 × 10** ^**−2**^	rs3217907	2.629 × 10^−2^	
		rs3217907	4406836	A	Additive	1.322 (1.087–1.608)	5.189 × 10^−3^	rs3217892	1.631 × 10^−1^	5.589 × 10^−3^
					Dominant	1.332 (1.017–1.744)	3.754 × 10^−2^	rs3217892	3.865 × 10^−1^	
					Recessive	1.708 (1.134–2.572)	1.035 × 10^−2^	REF^c^	REF	
12	IGF1	rs6214	102793569	T	Additive	1.158 (0.970–1.383)	1.040 × 10^−1^	—	—	1.086 × 10^−1^
					Dominant	1.474 (1.122–1.937)	5.371 × 10^−3^	rs3217892	2.353 × 10^−2^	
					Recessive	0.972 (0.727–1.299)	8.471 × 10^−1^	—	—	
15	IGF1R	rs11247380	99440731	G	Additive	1.145 (0.955–1.372)	1.431 × 10^−1^	—	—	1.562 × 10^−1^
					Dominant	0.954 (0.730–1.245)	7.274 × 10^−1^	—	—	
					Recessive	1.757 (1.224–2.524)	2.269 × 10^−3^	REF^c^	REF	
		rs2715417	99453047	T	Additive	1.148 (0.949–1.388)	1.554 × 10^−1^	—	—	1.798 × 10^−1^
					Dominant	0.997 (0.777–1.278)	9.778 × 10^−1^	—	—	
					Recessive	2.077 (1.292–3.340)	2.561 × 10^−3^	rs11247380	2.128 × 10^−1^	
		rs2684779	99406245	G	Additive	1.251 (0.991–1.580)	6.010 × 10^−2^	—	—	6.243 × 10^−2^
					Dominant	1.152 (0.877–1.512)	3.088 × 10^−1^	—	—	
					Recessive	3.621 (1.426–9.194)	6.789 × 10^−3^	rs11247380	7.206 × 10^−2^	
18	BCL2	rs7243091	60880562	A	Additive	1.080 (0.861–1.355)	5.058 × 10^−1^	—	—	5.205 × 10^−1^
					Dominant	1.255 (0.967–1.628)	8.719 × 10^−2^			
					Recessive	0.432 (0.228–0.815)	9.640 × 10^−3^	REF^c^	REF	

^a^The P values are based on logistic regression analysis and adjusted for age.

^b^The conditional P values are based on logistic regression analysis and adjusted for age and reference SNPs.

^c^The reference SNPs were applied to be adjusted in the conditional analysis.

^d^Only one SNP with the P < 0.05 was shown in additive model (for chromosome 3, 5, 6, 12, 18 and 22), dominant model (chromosome 3 and 22) and recessive model (chromosome 6 and 11).

^e^P for Cochran–Armitage trend test.

To confirm the association, we next performed the Cochran–Armitage trend test and conditional analysis. This analysis identified ten SNPs suggested to be associated with aggressive prostate cancer in additive and dominant models (rs603781, rs1050171, rs3217892, rs11231741, rs4705415, rs12643184, rs3217869, rs17035367, rs5757573 and rs11923427) and three loci in the additive and recessive model (rs3217869, rs9487729 and rs6828477) (Table 2 and Supplementary Table 1). Only one SNP (rs3217869 in *CCND2*) was shown to be significantly associated with aggressive prostate cancer after the analysis in the three models (Additive model: *P* = 4.653 × 10^−3^, OR = 1.511, 95% CI = 1.135–2.010; Dominant model: *P* = 5.341 × 10^−3^, OR = 6.907, 95% CI = 1.773–26.900; Recessive model: *P* = 2.156 × 10^−2^, OR = 1.435, 95% CI = 1.055–1.954), Cochran–Armitage trend test (*P* = 5.188 × 10^−3^) and conditional analysis (Additive model: *P* = 7.263 × 10^−3^; Dominant model: *P* = 8.938 × 10^−3^; Recessive model: *P* = 2.629 × 10^−2^) (Table 2).

We also tested the association of rs3217869 with aggressive prostate cancer in a Finnish cohort including 1729 aggressive and 693 non-aggressive cases. Consistently, rs3217869 A allele showed a tendency for risk in aggressive cases compared to non-aggressive prostate cancer though the results were not statistically significant (Dominant model: *P* = 0.273, OR = 1.116, 95% CI = 0.917–1.357; Recessive model: *P* = 0.221, OR = 1.144, 95% CI = 0.923–1.418). Together, the results from both cohorts suggest that the prostate cancer patients carrying rs3217869 A allele have a tendency to increase the risk for aggressive disease.

While only one locus was defined in the different tests, we also tried a further analysis to discover more potential loci. In this analysis, the loci identified in more than two tests were included. We tested whether these SNPs associated with the GS and clinical stages of prostate cancer. We found that the mutation of rs12643184 (*PDGF-C*) (Additive model: *P* = 3.612 × 10^−2^, OR = 1.374, 95% CI = 1.021–1.848; Dominant model: *P* = 2.094 × 10^−2^, OR = 1.484, 95% CI = 1.062–2.075) (Supplementary Table 1) was suggested to be the risk factor increasing the risk of aggressive prostate cancer (GS: mutation = 7.72 ± 1.45, wild type = 7.43 ± 1.71, *P* = 0.034) (Supplementary Table 2).

### Gene-based association analysis

After conducting the analysis with the SNPs available for the genes in the RTK/ERK pathway, we focused on all genes in the pathway and performed the gene-based analysis using the ARTP method^24^. This analysis revealed a marginal association between the RTK/ERK pathway genes including *BAD* (*P* = 0.058), *PDGF-D* (*P* = 0.051) and *CCND2* (*P* = 0.052) and aggressive prostate cancer.

### Functional annotation of newly-identified SNPs and genes including rs3217869/*CCND2*

**Figure 1:**
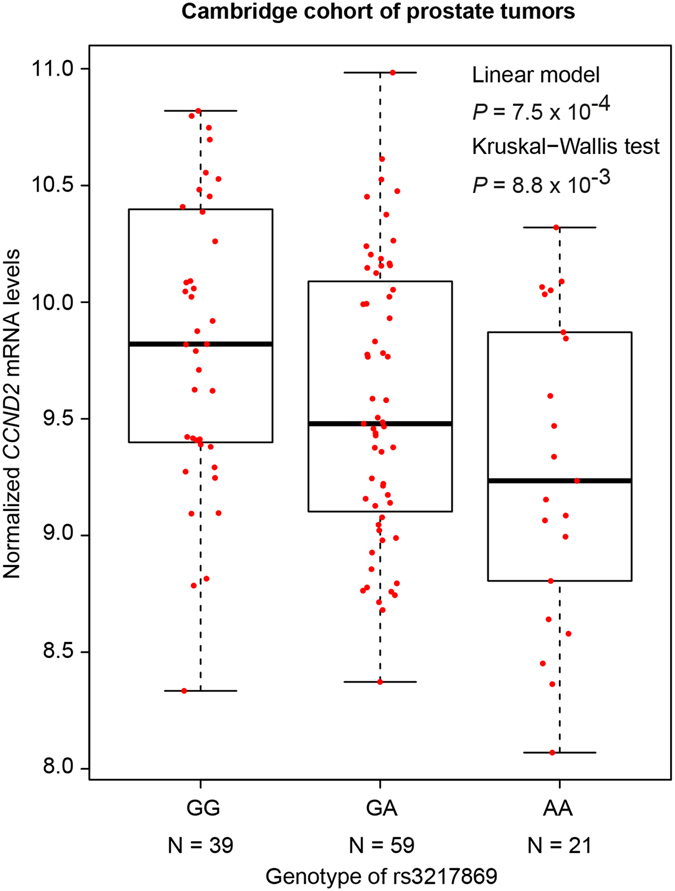
The association between rs3217869 genotype and *CCND2* expression in prostate tissue samples. Note that the risk A allele of rs3217869 for aggressive prostate cancer was significantly associated with decreased mRNA expression of *CCND2*. The *CCND2* mRNA levels were assessed by Illumina Expression BeadChip-based transcriptional profiling in a collection of 119 human prostate tissue samples^4, 25^. The *CCND2* mRNA expression data in prostate tumor tissues are displayed as the five number distribution (minimum, first quartile, median, third quartile, and maximum) according to rs3217869 genotype The *P* values were examined by linear regression model and Kruskal-Wallis H test, respectively.

This genetic study implicates the association of the SNPs rs3217869 (*CCND2*) and rs12643184 (*PDGF-C*), and the genes *BAD*, *PDGF-D* and *CCND2* in the RTK/ERK signaling pathway with aggressive prostate cancer. To gain insight into the underlying biological basis for the associated SNPs, we performed the expression quantitative trait loci (eQTL) analysis using array-based transcriptomics data derived from prostate tissues and the matched genotype information on 119 patients with prostate cancer^4, 25^. This analysis revealed a strong association of the risk allele A at rs3217869 with lower mRNA levels of *CCND2* (Fig. 1) but not with other genes within 2 Mb of rs3217869 (Supplementary Table 3), suggesting that *CCND2* is a plausible causative gene for aggressive prostate cancer. In contrast, no significant association was found for rs12643184 (*PDGF-C*) within this eQTL data set.

**Figure 2:**
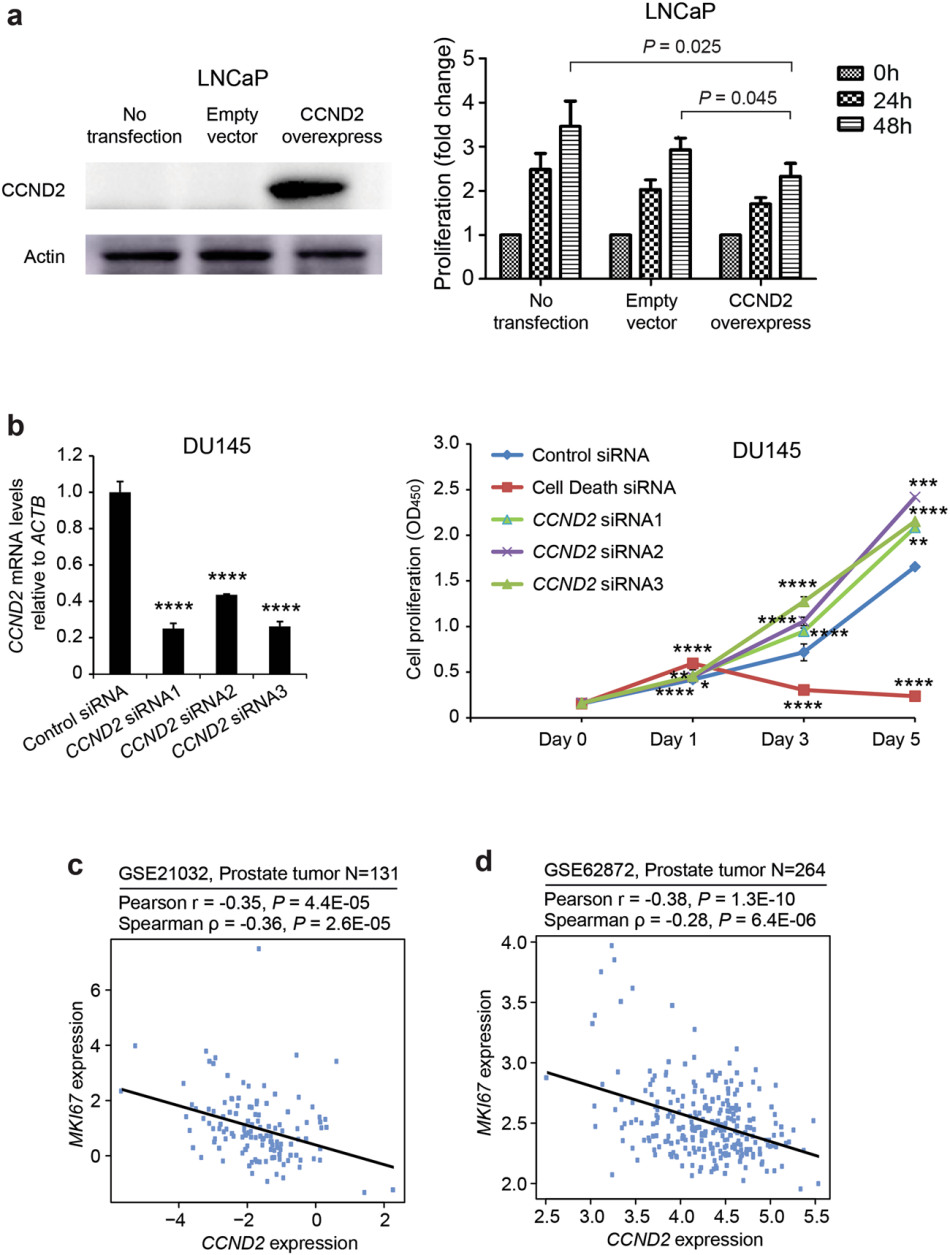
CCND2 plays a role in prostate cancer cell proliferation. (**a**) Ectopic expression of CCND2 in LNCaP inhibits its cellular proliferation. Left: Measurement of CCND2 overexpression by Western blot. Note that the three lanes represent LNCaP without transfection, LNCaP transfected with empty vector and LNCaP transfected with CCND2 expression plasmids, respectively. Right: The results of MTT in LNCaP cells upon CCND2 overexpression. Comparing to controls (no transfection or transfection with empty vector), the proliferations of LNCaP cells were significantly inhibited. The absorbance at 570 nm was measured with EPOCH2 Microplate Readers (BioTek), mean ± s.d. of four independent experiments. (**b**) Knockdown of *CCND2* in DU145 promotes its cellular proliferation. Left panel: The knockdown efficiency of siRNAs against *CCND2* were measured by quantitative real-time PCR. Right panel: Knockdown of *CCND2* increases cell proliferation of prostate cancer DU145 cell line measured by XTT colorimetric assay (absorbance at 450 nm (OD450); mean ± s.d. of three independent experiments. *P < 0.05, **P < 0.01, ***P < 0.001, ****P < 0.0001. In (**a** and **b**), the P values were assessed by the two-tailed Student’s t test. (**c**,**d**) Expression correlation of *CCND2* with *MKI67* in human prostate tumor samples. Scatter plot showing the inverse correlation between *CCND2* and *MKI67* expression in two independent cohorts of prostate tumors (n = 131 and n = 264, respectively)^30, 31^.

*CCND2* was previously reported to be frequently methylated, with loss of its expression in prostate cancer^29^, suggesting a potential role as a tumor suppressor. Consistent with this, we found that overexpression of *CCND2* in prostate cancer cell line LNCaP significantly inhibited its proliferation (Fig. 2a). Moreover, short interfering RNA (siRNA)-mediated knockdown of *CCND2* in prostate cancer cells DU145 greatly promoted cell growth and viability compared to the cells with negative control siRNAs (Fig. 2b). In contrast, we observed a slight change in PC3 cells upon siRNA-mediated knockdown of *CCND2* with similar cell proliferation experiments (Supplementary Fig. 1), indicating that *CCND2*-directed cell growth inhibition may be cell-context dependent. In addition, in multiple large cohorts of clinical prostate cancer samples^30, 31^, we observed significantly inverse correlations between the mRNA expression of *CCND2* and *MKI67* (Fig. 2c and d), the latter coding Ki67 antigen, a recognized indicator of cellular proliferation.

**Figure 3:**
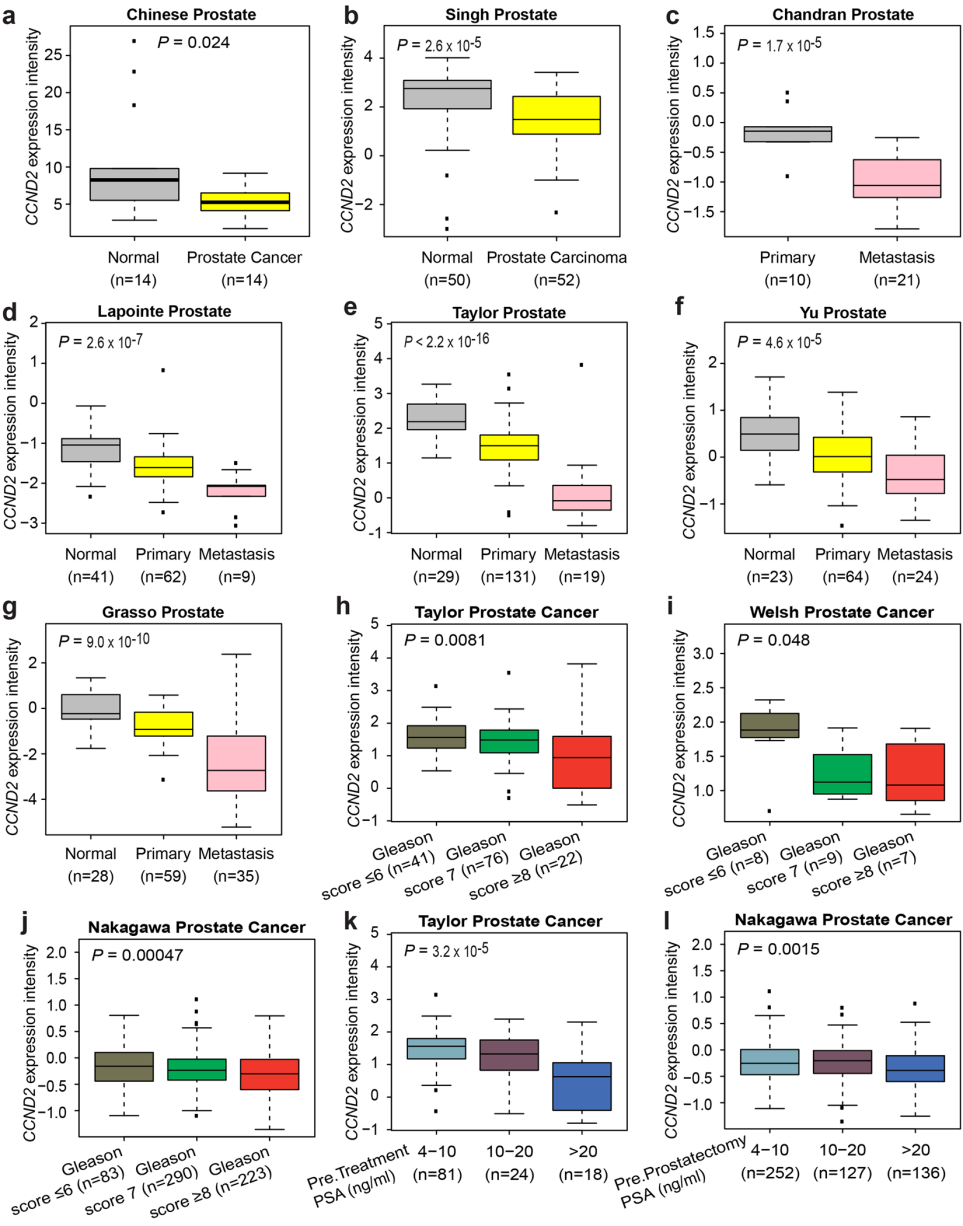
*CCND2* mRNA expression is strikingly downregulated in metastatic human prostate cancer and its underexpression correlates with disease aggressiveness. (**a**–**g**) *CCND2* transcript levels are greatly decreased in primary and metastatic prostate tumor tissues in several clinical data sets^30, 32–37^, including a cohort of prostate samples of Chinese men^32^. Normal, benign prostate gland. Primary, primary human prostate cancer. Metastasis, metastatic prostate samples. (**h**–**j**) Decreased *CCND2* expression correlates with prostate tumor progression to high Gleason score in multiple cohorts of patients with prostate cancer^28, 30, 38^. (**k**,**l**) *CCND2* underexpression markedly correlates with elevated serum PSA levels in the Taylor *et al*.^30^ and Nakagawa *et al*.^28^ clinical data sets. PSA is a diagnostic marker of prostate gland malignancy. Pre.Treatment or Pre.Prostatectomy PSA refer to the PSA level prior to radical prostatectomy. In (**a**), *CCND2* expression intensity was determined by RNA sequencing^32^; (**b**–**l**), *CCND2* expression intensity is log2 median-centered intensity as reported in Rhodes, D. R. *et al*.^26^.

**Figure 4:**
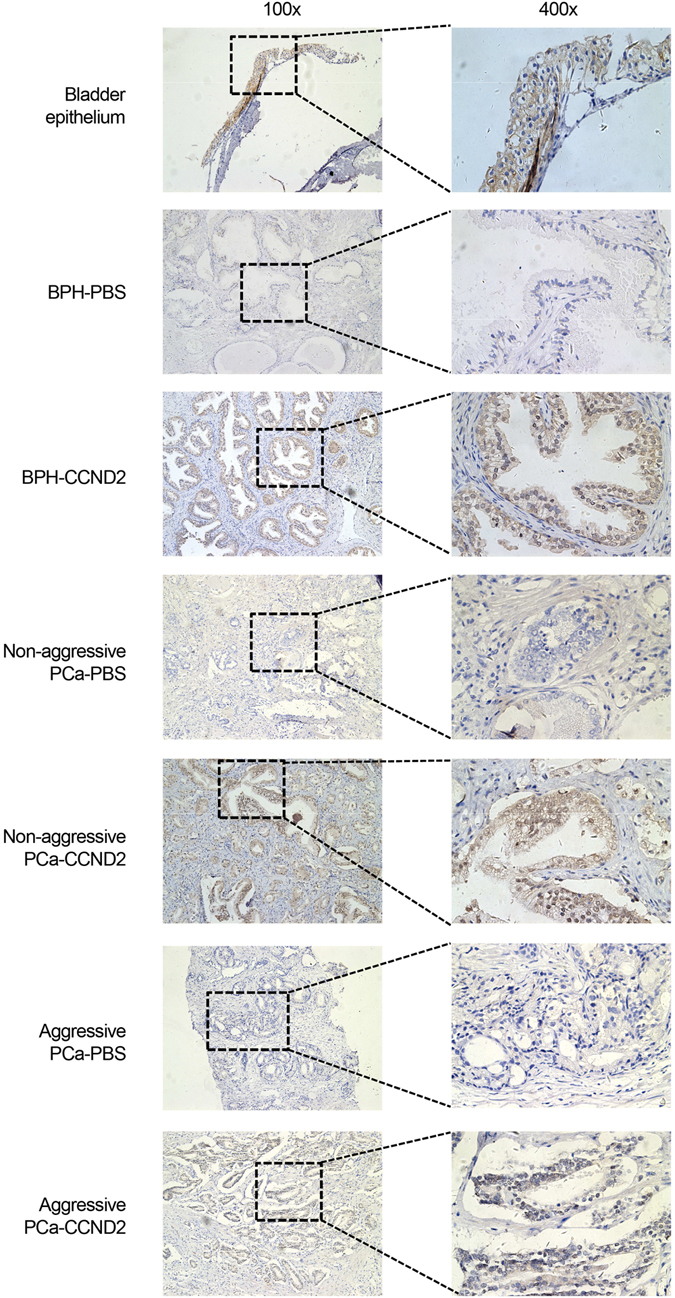
The immunohistochemistry staining analysis of CCND2 expression in the bladder epithelium, benign prostatic hyperplasia (BPH), aggressive and non-aggressive prostate cancer specimens (left: 100x and right: 400x magnification). Note that, according to BioGPS (http://biogps.org/#goto=genereport&id=12566&show_dataset=BDS_00007), the CCND2 was highly expressed in bladder epithelium cells. So, bladder epithelium was used as the positive control with strong staining signal using a CCND2-specific antibody. Staining with no antibody (PBS) as negative control. Note that, in contrast to BPH and non-aggressive prostate cancer, aggressive specimens showed less staining signal of CCND2 expression. The boxes around the area in the 100x images indicate the regions highlighted in the 400x images.

We next investigated the status of the associated genes in human prostate tissues by analyzing the expression levels of *BAD*, *CCND2*, *PDGF-C*, and *PDGF-D* in tens of independent cohorts of clinical expression profile data sets^28, 30, 32–52^. The results showed that the mRNA levels of *BAD*, *CCND2* and *PDGF-D* were differentially expressed in prostate tumor tissues and normal prostate gland (Figs 3a–g and Supplementary Figs 2a–j, 3a–f and 4a–d). *BAD* was significantly upregulated in primary and metastatic prostate tumors compared to matched normal tissues^30, 34, 35, 37–39^ (Supplementary Fig. 3a–f). *PDGF-D* showed a strong tendency to be highly expressed in metastatic prostate cancer samples^34, 40, 45, 51^ (Supplementary Fig. 4a–c). In particular, the transcriptional levels of *CCND2* were significantly lower in prostate tumors than in normal tissues, and the results from one cohort of Chinese^32^ and other sixteen independent clinical data sets^30, 32–47^ with a total amount of 1,083 samples showed that *CCND2* was markedly underexpressed upon tumor progression to advanced stage and metastasis (Fig. 3a–g and Supplementary Fig. 2a–j). We also investigated the expression of CCND2 at protein levels using immunohistochemistry staining on prostatic tissue specimens of Chinese men. This analysis revealed that loss of CCND2 expression appeared to be in aggressive prostate cancer (Fig. 4). While all the Benign Prostatic Hyperplasia (BPH, n = 3) and non-aggressive prostate cancer (n = 4) showed positive CCND2 staining, only 50% of aggressive prostate cancer (n = 5) appeared to be positive with CCND2 expression (see **Methods**), consistent with one previous study^53^ showed that CCND2 proteins were frequently lost in a cohort of prostate cancer specimens by immunohistochemistry staining analysis.

Additionally, we analyzed prostate cancer expression data from men with clinical records of Gleason score and prostate-specific antigen (PSA) levels^28, 30, 38, 40^, the indicators of prostate cancer aggressiveness, finding that *CCND2* mRNA levels were significantly lower in tumors with high Gleason score and elevated PSA levels (Fig. 3h–l and Supplementary Fig. 2k–m). In contrast, higher transcript levels of *BAD*, *PDGF-C* and *PDGF-D* appeared to correlate with high Gleason score and PSA levels^30, 35, 37, 40, 49–52^ (Supplementary Figs 3g–l and 4e–h and 5), suggesting that these three genes may possess oncogenic functions whereas *CCND2* is a potential tumor and metastasis suppressor in prostate cancer.

### *CCND2* indicates prognostic potential in prostate cancer

**Figure 5:**
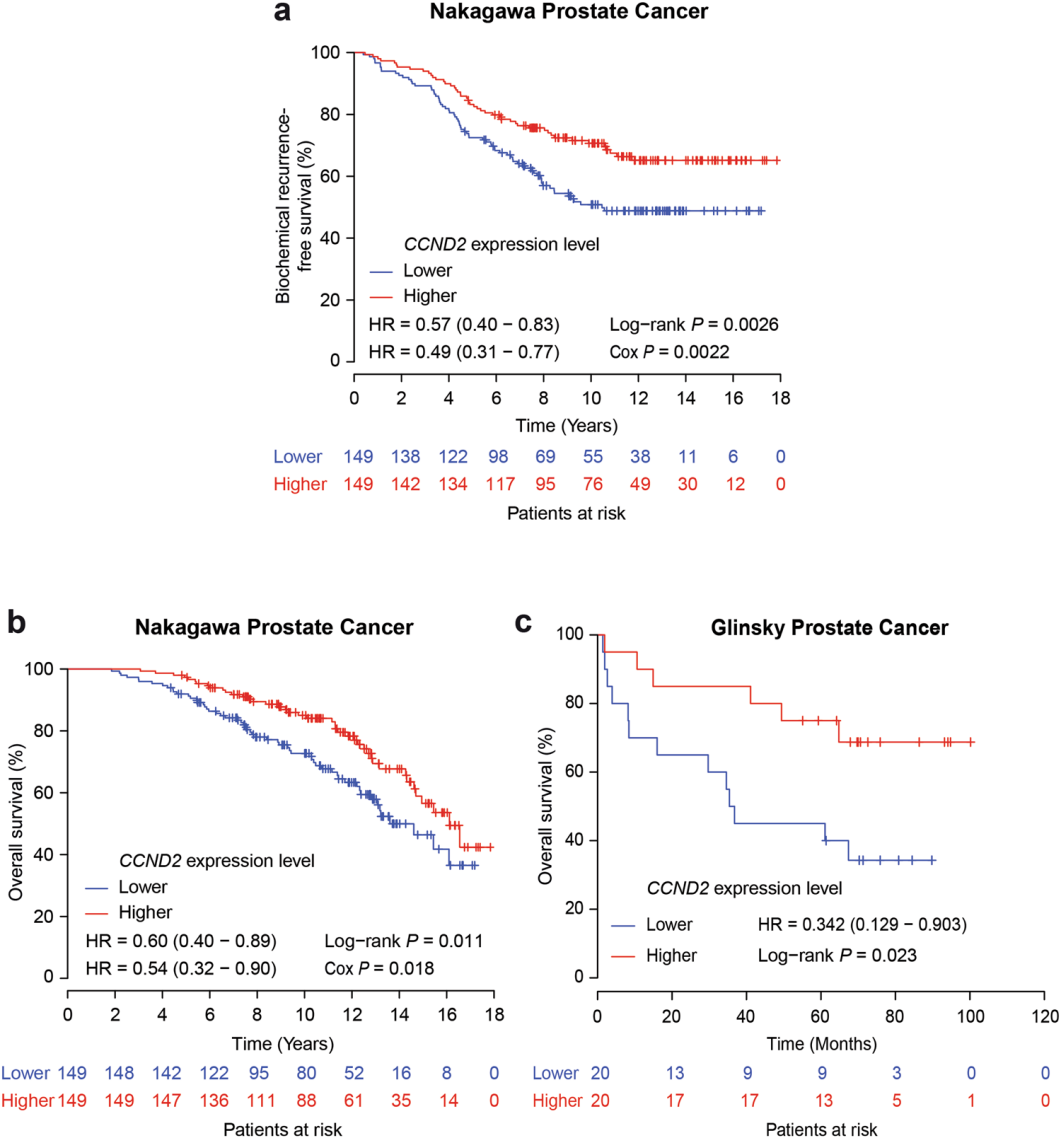
*CCND2* downregulation correlates with poor prognosis of the patients with prostate cancer. Kaplan-Meier curves and estimates of the risk for biochemical recurrence (**a**), and overall survival (**b**) in a large group of prostate cancer patients^28^ with higher (top 50%; n = 149) or lower (bottom 50%; n = 149) expression levels of *CCND2*, and (**c**) in another independent cohort^54^. Overall, the patients with tumors expressing lower levels of *CCND2* show elevated risk of biochemical relapse and decreased overall survival. The number of patients in each group at every 2-year (**a**,**b**) and (**c**) 20-month intervals was indicated. The *P* value was calculated by a Log-rank test (**a**–**c**) and Cox regression model (**a**,**b**), respectively.

Among these analyses, *CCND2* indicates the most prominent association with aggressive prostate cancer, supporting *CCND2* as the most plausible causative gene through our integrated genetic association, follow-up eQTL and functional studies. We thus investigated the potential clinical relevance of *CCND2* in prostate cancer in a large collection of prostate cancer cases^28^. Using the Kaplan-Meier analysis, we examined the association of *CCND2* expression with clinical variables indicating prostate cancer aggressiveness, and found that the time to postoperative PSA recurrence and for overall survival was significantly shorter in the patient group with lower expression levels of *CCND2* (*P* < 0.05; Fig. 5a and b), the latter association was also observed to be significant in another independent cohort of prostate cancer^54^ (*P* = 0.023; Fig. 5c). Notably, *CCND2* downregulation also showed a strong tendency correlation with increased risk of biochemical relapse in an additional collection of prostate tumors^30^ (Supplementary Fig. 6a). In addition, to address the question of whether *CCND2* levels have predictive values for low- and high-risk cases, we subdivided a large cohort of prostate cancer^55, 56^ by Gleason score and compared the frequency of postoperative biochemical recurrence. This analysis revealed a trend of *CCND2* levels in predicting biochemical recurrence in prostate cancer patients with a Gleason score of 7 (intermediate risk, n = 246; Supplementary Fig. 6b), but not for the cases with a Gleason score of 6 (low risk, n = 44; Supplementary Fig. 6c) or the very high-risk patients (Gleason score ≥8, n = 201; Supplementary Fig. 6d), suggesting that *CCND2* is a potential biomarker to distinguish the patients that are likely to recur in the patient group at intermediate risk.

To further assess the prognostic potential of *CCND2* in prostate cancer and determine the relative dependency of PSA recurrence and overall survival, we performed multivariate analysis, including Gleason score, PSA levels, age and clinical T stage. This analysis revealed that the mRNA level of *CCND2* was determined to be an independent predictor of biochemical recurrence and overall survival (*P* < 0.05; Fig. 5a and b). These results demonstrate a clear association of *CCND2* downregulation with prostate cancer development, progression and poor prognosis in patients. Together with our genetic and eQTL evidence of the strong association between rs3217869 risk genotype and reduced *CCND2* expression, this study identified *CCND2* as a plausible causative gene and potential metastatic tumor suppressor underlying genetic predisposition and disease progression to aggressive prostate cancer.

## Discussion

Aggressive prostate cancer indicates poor prognosis for the disease development. In our previous work^10–12^, a potential association was revealed between prostate cancer risk and the RTK/ERK pathway. Considering the disease development, a study on the association between aggressive prostate cancer and this pathway was needed, which could implicate the RTK/ERK pathway in the progression of prostate cancer and shed new insight into the targeted therapy for prostate cancer. Through a comprehensive analysis, we identified two SNPs rs3217869 in *CCND2* and rs12643184 in *PDGF-C* suggested to be important. In addition, we observed a marginal association of three genes including *CCND2* with aggressive prostate cancer.

The present study was conducted using the ChinaPCa data. At the SNP-level, the tag-SNPs in all 40 genes within the pathway were collected. After the association analysis, the SNPs with *P* < 0.05 were selected for the next analysis. As for the gene-based analysis, the ARTP method was applied. We identified three genes *BAD*, *CCND2*, and *PDGF-D* with a marginal association with aggressive prostate cancer. Combined with the results of SNP-level analysis, we presented two loci to be associated with prostate cancer aggressiveness. One is rs3217869 in *CCND2* that was discovered by various model analyses, and suggested to be a risk factor (rs3217869 A allele) for aggressive prostate cancer. Consistently, this risk tendency of rs3217869 for aggressive prostate cancer was also observed in a large cohort of Finnish prostate cancer patients comprising 1729 aggressive and 693 non-aggressive cases. A slightly risk effect of allele A at rs3217869 was identified. This is in agreement with the eQTL analysis indicating a strong association of the allele A at rs3217869 with decreased transcript levels of *CCND2*, a potential tumor suppressive gene in prostate cancer and strongly implicating tumor progression. The other locus is rs12643184 (*PDGF-C*). rs12643184 was indicated as a risk factor related to the progression of prostate cancer with OR >1 in the additive and dominant models. Meanwhile, based on the clinical information (GS and clinical stage), it was suggested that the mutation of rs12643184 was associated with increased GS in the aggressive prostate cancer, consistent with the association result as described above.

As for the associated gene, rs3217892 is within *CCND2*, a member of D-type cyclins (CCND) that are known to be responsible for G1 phase progression. There are three D-type cyclins (CCND1, CCND2 and CCND3) involved in the regulation of transition from G1 to S during the cell cycle^57, 58^. Despite the fact that the overexpression of *CCND2* has been reported in some cancers such as gastric cancer^16, 59, 60^, our analyses showed a striking downregulation of *CCND2* and its association with disease progression and poor prognosis in multiple independent cohorts of prostate cancer, suggesting that *CCND2* is a potential tumor suppressor in prostate carcinoma. Consistently, our results together with the previous reports^53, 61^ showed that restoration of CCND2 expression inhibited the proliferation of prostate cancer cells, and *CCND2* knockdown accelerated prostate cancer cell proliferation. This is in line with our eQTL analysis that the risk allele A at rs3217869 is significantly associated with *CCND2* downregulation, indicating a potential causal role of *CCND2* underlying prostate cancer susceptibility and severity. In addition, a striking downregulation of *CCND2* observed in this study may be partially explained by a previous report of promoter methylation of *CCND2* in prostate cancer and its association with clinicopathological features of poor prognosis^29^.

The other SNP, rs12643184 is in *PDGF-C*, a member of the platelet-derived growth factor (*PDGF*) family. This family consists of four different polypeptide chains (PDGF-A, -B, -C and -D) that were mainly combined with two receptors (PDGFR-α and β) to perform the biological functions^62^. Activated PDGF-C and PDGF‑D are a high affinity ligand for PDGFR‑α and PDGFR-β homodimers, respectively^63^. Recent studies suggested that these two genes were associated with tumor progression and angiogenesis^64, 65^. PDGF-C was suggested to take part in the modulation of human melanoma cell invasiveness^66^. In addition, blockade of PDGF-C could inhibit pathological angiogenesis^67^, with the potential to be applied in cancer therapy. We thus hypothesized that the PDGF-C might be important in the metastasis and progression of prostate cancer to some extent. In line with this, we found that PDGF-C indicated a tendency to be highly expressed in advanced prostate tumors with high Gleason score and PSA levels.

PDGF-D consists of a two-domain structure with an N-terminal CUB domain (for complement C1r/C1s, Uegf, Bmp1), and a C-terminal PDGF/vascular endothelial growth factor domain^68^. Activated PDGF-D binds to its cognate receptor PDGFR-β inducing the removal of CUB domain and contributing to the development and progression of diseases^69, 70^. PDGF-D has been revealed with the potential oncogenic activity in the development of prostate cancer^71^. Consistently, we observed a high tendency of *PDGF-D* upregulation in the metastatic prostate tumors and the tumors indicating high Gleason score and PSA levels. Moreover, in metastatic prostate cancer, PDGF-D was reported to be a regulator of osteoclastic differentiation that is critical for the establishment of skeletal metastatic deposits in prostate cancer patients^72^.

There are limited studies to link the association between *BAD* gene and aggressive prostate cancer. In support of the role for *BAD* in prostate cancer progression, one recent study^73^ suggested that BAD activation through the JNK and PI3K/Akt pathways contributed to prostate tumor growth under chronic hypoxia. In addition, a study in non-small cell lung cancer^74^ showed that the loss of *BAD* independently predicted poor prognosis. In addition to this, our results showed a markedly increased expression of *BAD* in advanced prostate cancer samples, further supporting this genetic identification of the association of *BAD* with aggressive prostate cancer.

In summary, our combined SNP- and gene-based analyses revealed the association of two novel loci (rs3217869/*CCND2* and rs12643184/*PDGF-C*), and three genes (*BAD*, *PDGF-D* and *CCND2*) in the RTK/ERK signaling pathway with aggressive prostate cancer. Our eQTL study together with a comprehensive analysis of tens of independent clinical data sets highlight the potential role of *CCND2* as a plausible causative gene with metastatic tumor suppressive activity in aggressive prostate cancer, providing promising clues on risk stratification and targeted therapy in men with advanced prostate cancer.

## Electronic supplementary material

Supplementary Information
