# Supplementary material for: Genetic association analysis of the RTK/ERK pathway with aggressive prostate cancer highlights the potential role of CCND2 in disease progression
Source: Sci Rep. 2017 Jul 3;7:4538. doi: 10.1038/s41598-017-04731-4 (PMC5495790; doi:10.1038/s41598-017-04731-4)
Supplement: Supplementary file 1 — Supplementary Information [file 41598_2017_4731_MOESM1_ESM.pdf]

Supplementary Information

Genetic association analysis of the RTK/ERK pathway with aggressive prostate cancer highlights the potential role of *CCND2* in disease progression

Yang Chen[1,2,3]*, Qin Zhang[4,5]*, Qiuyan Wang[1,3]*, Jie Li[1,6]*, Csilla Sipeky[7], Jihan Xia[4,5], Ping Gao[4,5], Yanling Hu[1,3,8], Haiying Zhang[1,3], Xiaobo Yang[1,3], Haitao Chen[9], Yonghua Jiang[1,3], Yuehong Yang[4,5], Ziting Yao[1,3], Yinchun Chen[1,3], Yong Gao[1, 3], Aihua Tan[1,3], Ming Liao[1,3], Johanna Schleutker[7,10], Jianfeng Xu[1,3,9,11], Yinghao Sun[12], Gong-Hong Wei[4,5]† & Zengnan Mo[1,2,3]†

*These authors contributed equally to this work.

†Correspondence and requests for materials should be addressed to Z.M. (email: zengnanmo@hotmail.com) or G.-H.W. (email: gonghong.wei@oulu.fi)

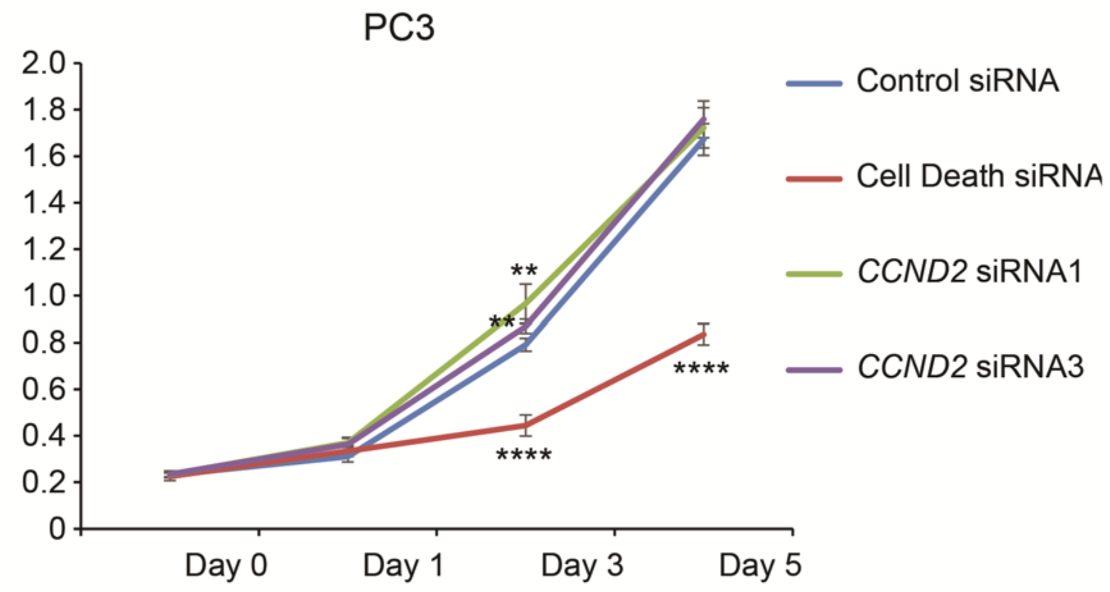

Supplementary Figure 1. The role of *CCND2* in prostate cancer cell growth. Knockdown of *CCND2* in PC3 slightly promotes its cellular proliferation. The two most efficient siRNAs against *CCND2* (see Figure 2b, left) were selected for cell proliferation assays in PC3 cells to evaluate the role of *CCND2* in prostate cancer cell growth. A slight increase in cell proliferation of prostate cancer PC3 cell line upon knockdown of *CCND2* was measured by XTT colorimetric assay (absorbance at 450nm (OD450); mean ± s.d. of three independent experiments. **$P < 0.01$, ****$P < 0.0001$. The P values were examined by the two-tailed Student's t test.

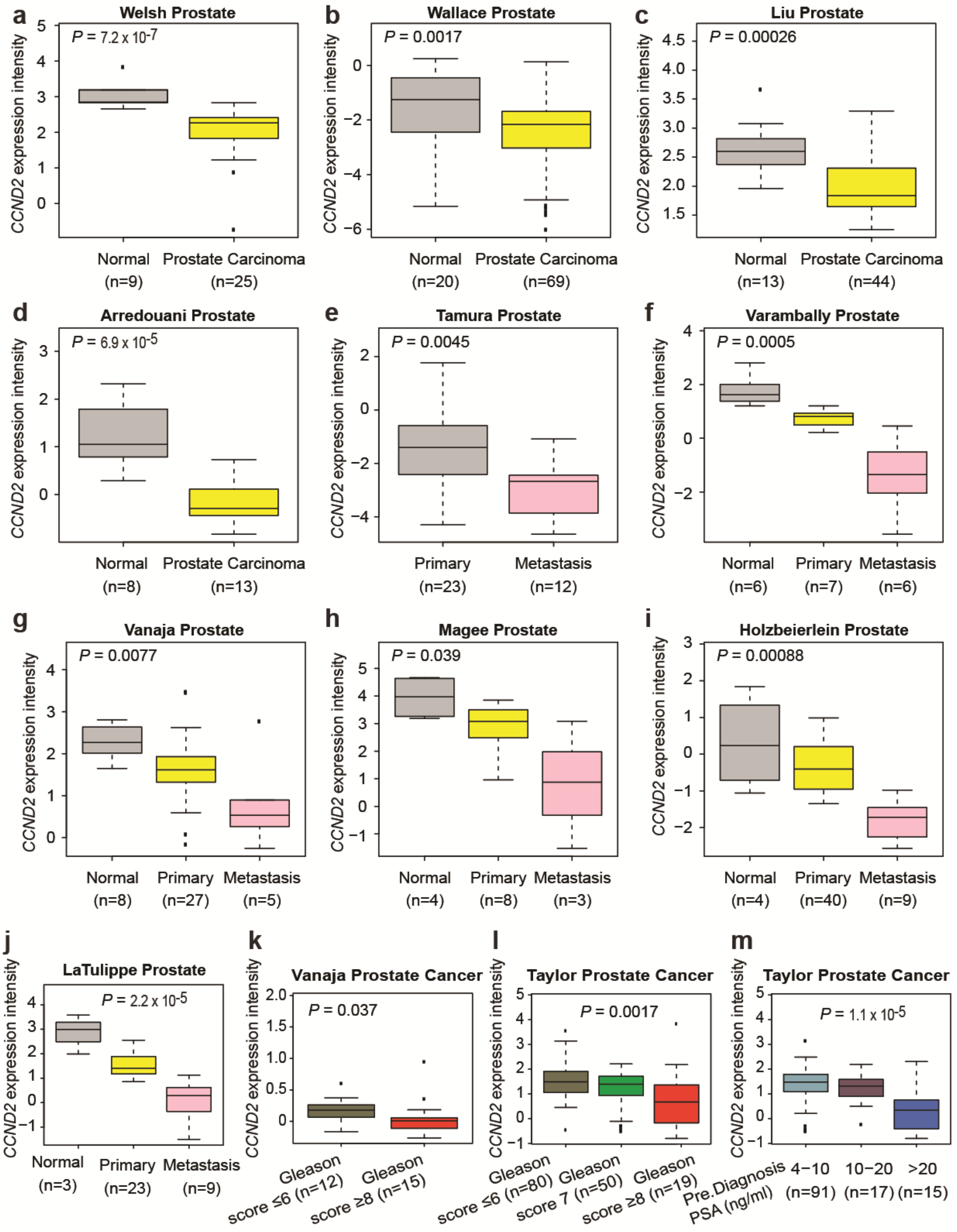

Supplementary Figure 2. *CCND2* downregulation in human prostate cancer correlates with tumor progression to advanced stages. (**a-j**) *CCND2* expression levels are strikingly downregulated in primary and metastatic prostate tumor tissues in ten independent clinical data sets[38-47]. Normal, benign prostate gland. Primary, primary human prostate cancer. Metastasis, metastatic prostate samples. (**k,l**) *CCND2* downregulation

correlates with prostate tumor progression to high Gleason score in two independent cohorts of prostate cancer patients[30,40]. (**m**) *CCND2* underexpression markedly correlates with elevated serum PSA levels in the Taylor et al. clinical data set[30]. PSA is a diagnostic marker for prostate cancer. Pre.Diagnosis PSA: PSA level at diagnosis. In **a-m**, *CCND2* expression intensity is log2 median-centered intensity as described in Oncomine database[26].

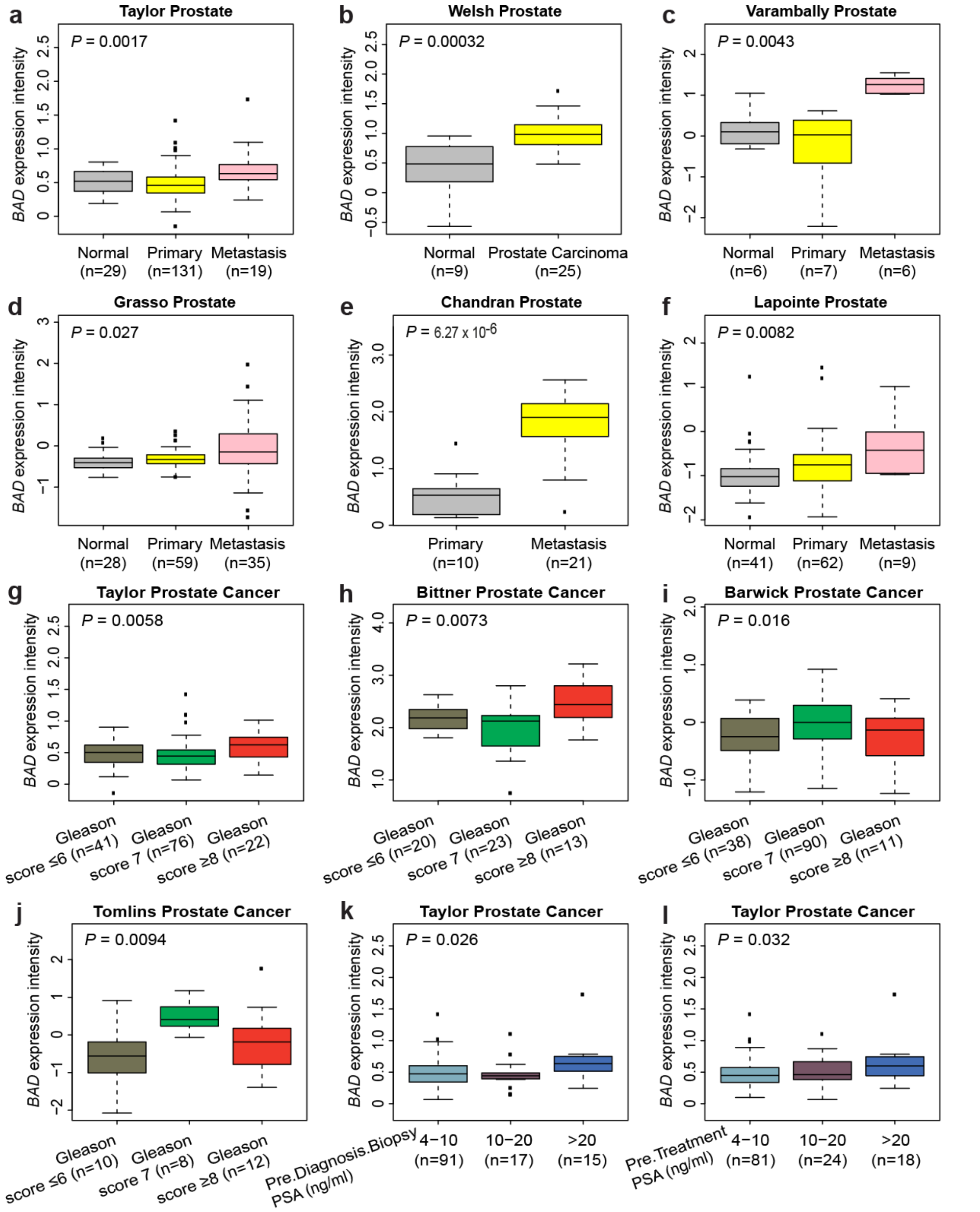

Supplementary Figure 3. *BAD* upregulation in human prostate cancer correlates with disease progression and aggressiveness. (**a-f**) *BAD* transcript levels are strikingly upregulated in primary and metastatic human prostate tumor tissues in several clinical data sets[30,34,35,37-39]. Normal, benign prostate gland. Primary, primary human prostate cancer. Metastasis, metastatic prostate samples. (**g-j**) Increased *BAD* expression correlates with prostate tumor progression to high Gleason score in multiple cohorts of patients with prostate cancer[30,50,51]. (**k,l**) *BAD* upregulation highly correlates with elevated serum PSA levels in the Taylor et al. clinical data set[30]. Pre.Diagnosis PSA indicates the PSA level at diagnosis. Pre.Treatment PSA refers to the PSA level prior to radical prostatectomy. In **a-l**, *BAD* expression intensity is log2 median-centered intensity as presented in Oncomine database[26].

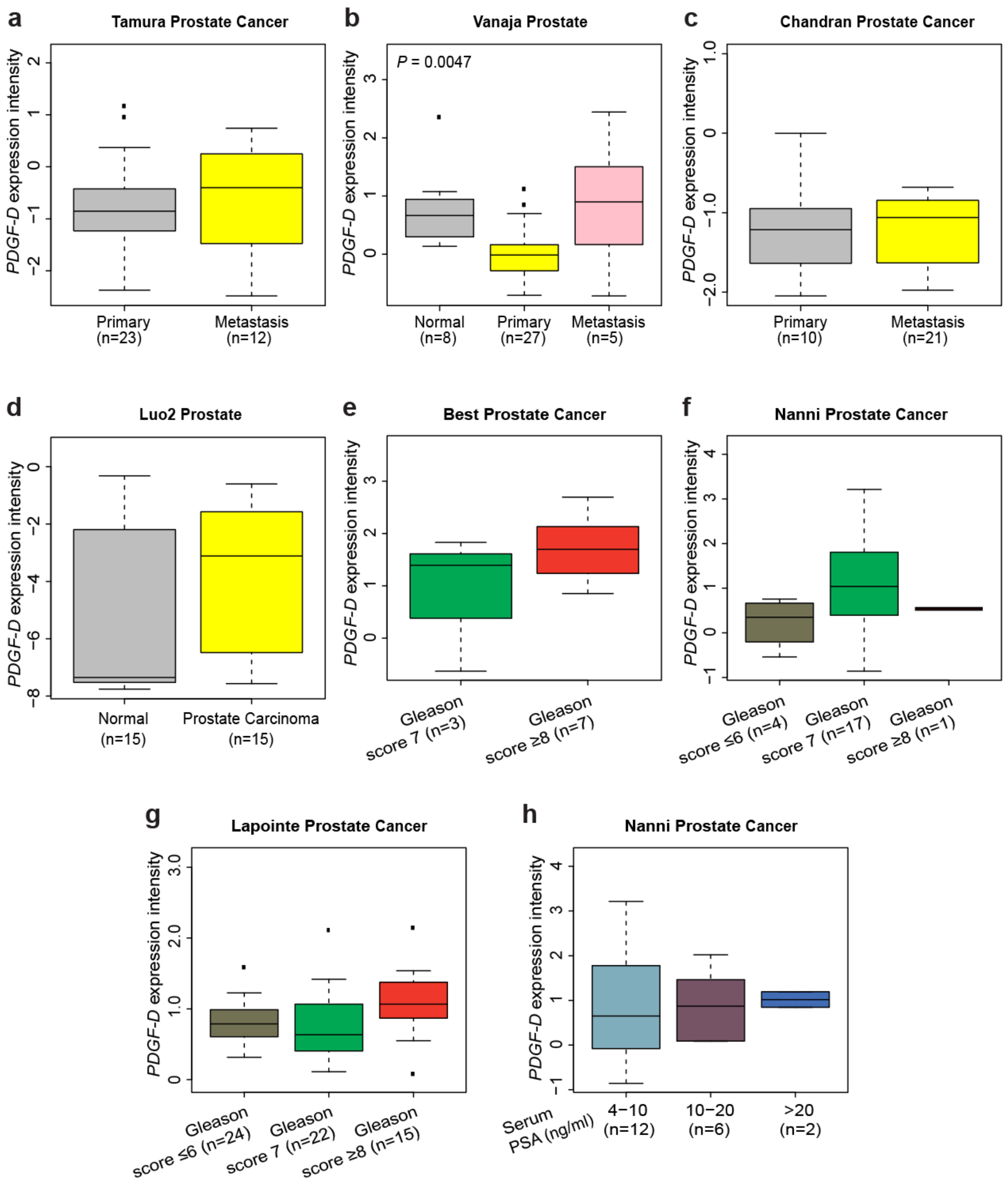

Supplementary Figure 4. Higher expression levels of *PDGF-D* show strong tendency in the correlation with human prostate cancer progression and aggressiveness. (**a-d**) *PDGF-D* is highly expressed in primary and metastatic human prostate tumor tissues compared with normal prostate gland[34,40,44,48]. Normal, benign prostate gland. Primary, primary human prostate cancer. Metastasis, metastatic prostate samples. (**e-h**) Higher mRNA levels of *BAD* correlates with prostate tumor progression to high Gleason score in several cohorts of prostate cancer patients[35,49,52], and elevated serum PSA levels in the Nanni prostate cancer data set[43]. In **a-h**, *BAD* expression intensity is log2 median-centered intensity as previously reported[26].

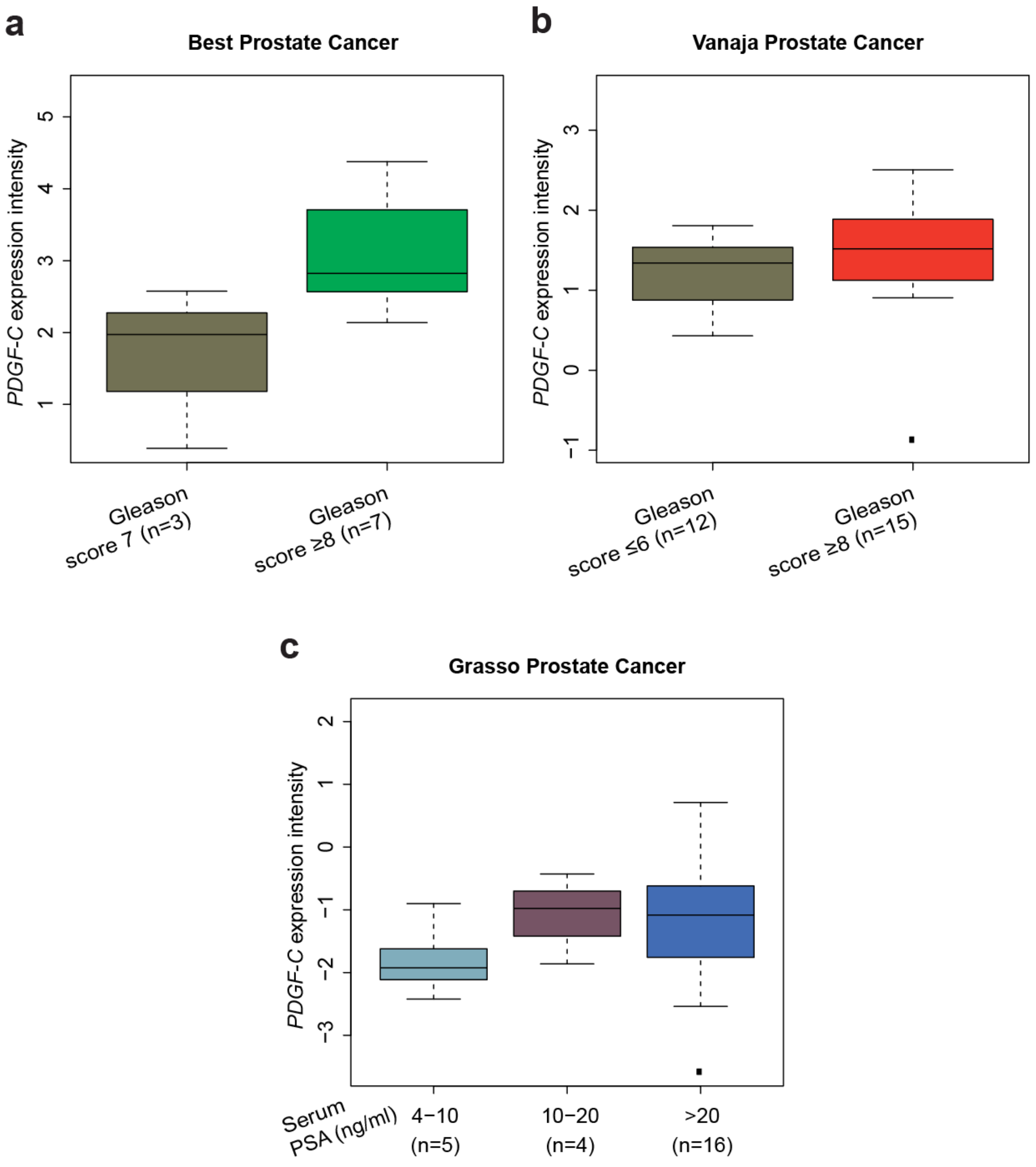

Supplementary Figure 5. *PDGF-C* expression levels are higher in human prostate cancer samples with elevated Gleason score (**a,b**) and serum PSA levels (**c**) in several independent data sets[37,40,49]. *PDGF-C* expression intensity shown as log2 median-centered intensity as previously described[26].

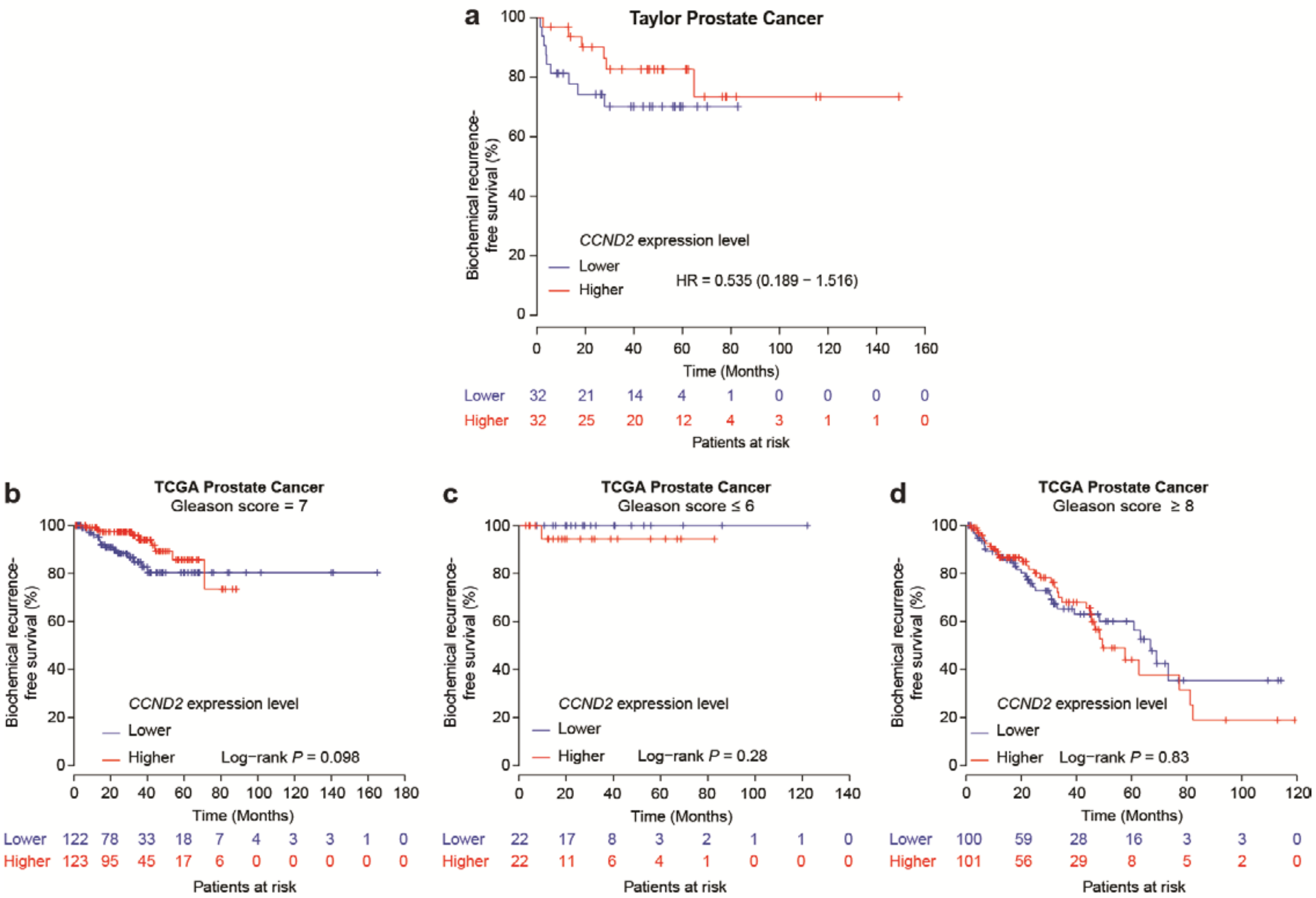

Supplementary Figure 6. *CCND2* downregulation correlates with increased risk of biochemical recurrence in a cohort of prostate cancer patients[30] (**a**) and a subgroup of cases with Gleason score of 7[55] (**b**), but in the subgroups of Gleason score ≤ 6 (**c**) or ≥ 8 (**d**). The number of patients at risk in each group at every 20-month interval was indicated. *P* value assessed by a Log-rank test.

CHR	Gene	SNP	BP	A1	Model	OR (95% CI)	P[a]	Conditional SNP	Conditional P value[b]	P[e]
3	RAF1	rs11923427	12663835	G	Additive	0.551 (0.311-0.977)	4.145×10^{-2}	No_REF[d]	No_REF	3.785×10^{-2}
					Dominant	0.531 (0.289-0.977)	4.198×10^{-2}	No_REF[d]	No_REF	
					Recessive	0.378 (0.024-6.059)	4.917×10^{-1}	-	-	
4	KDR	rs6828477	55966801	C	Additive	0.762 (0.618-0.939)	1.069×10^{-2}	REF[c]	REF	1.182×10^{-2}
					Dominant	0.758 (0.591-0.973)	2.968×10^{-2}	rs12643184	6.093×10^{-2}	
					Recessive	0.564 (0.323-0.987)	4.482×10^{-2}	rs2255355	4.013×10^{-2}	
		rs3816584	55964565	G	Additive	1.208 (1.011-1.443)	3.772×10^{-2}	rs6828477	2.313×10^{-1}	3.525×10^{-2}
					Dominant	1.263 (0.967-1.650)	8.635×10^{-2}	-	-	
					Recessive	1.313 (0.954-1.807)	9.437×10^{-2}	-	-	
		rs1531289	55955232	T	Additive	0.836 (0.670-1.043)	1.1280×10^{-1}	-	-	1.233×10^{-1}
					Dominant	0.872 (0.676-1.126)	2.939×10^{-1}	-	-	
					Recessive	0.499 (0.260-0.958)	3.661×10^{-2}	rs2255355	2.540×10^{-2}	
4	EGF	rs6818260	110887254	C	Additive	0.758 (0.599-0.960)	2.126×10^{-2}	rs6828477	2.129×10^{-2}	2.615×10^{-2}
					Dominant	0.760 (0.583-0.991)	4.266×10^{-2}	rs12643184	6.997×10^{-2}	
					Recessive	0.509 (0.234-1.110)	8.958×10^{-2}	-	-	

		rs7677295	110856192	T	Additive	0.768 (0.605-0.974)	2.935×10^{-2}	rs6828477	3.091×10^{-2}	3.551×10^{-2}
					Dominant	0.778 (0.596-1.016)	6.531×10^{-2}	-	-	
					Recessive	0.476 (0.216-1.049)	6.551×10^{-2}	-	--	
		rs881878	110836048	A	Additive	1.612 (1.043-2.491)	3.154×10^{-2}	rs6828477	3.991×10^{-2}	3.592×10^{-2}
					Dominant	1.583 (1.011-2.480)	4.475×10^{-2}	rs12643184	5.571×10^{-2}	
					Recessive	NA	9.985×10^{-1}	-	-	
		rs6533485	110927563	G	Additive	1.229 (1.002-1.509)	4.800×10^{-2}	rs6828477	4.261×10^{-2}	4.644×10^{-2}
					Dominant	1.230 (0.951-1.591)	1.146×10^{-1}	-	-	
					Recessive	1.588 (0.945-2.669)	8.062×10^{-2}	-	-	
4	PDGF-C	rs17035367	157786225	G	Additive	0.708 (0.517-0.969)	3.103×10^{-2}	rs6828477	3.164×10^{-2}	2.418×10^{-2}
					Dominant	0.681 (0.488-0.950)	2.375×10^{-2}	rs12643184	4.682×10^{-2}	
					Recessive	0.915 (0.177-4.739)	9.154×10^{-1}	-	-	
		rs12643184	157848474	T	Additive	1.374 (1.021-1.848)	3.612×10^{-2}	rs6828477	3.464×10^{-2}	3.566×10^{-2}
					Dominant	1.484 (1.062-2.075)	2.094×10^{-2}	REF[c]	REF	
					Recessive	1.130 (0.444-2.877)	7.985×10^{-1}	-	-	
		rs6822796	157887357	T	Additive	1.227 (1.005-1.497)	4.459×10^{-2}	rs6828477	4.359×10^{-2}	4.243×10^{-2}
					Dominant	1.226 (0.954-1.576)	1.116×10^{-1}	-	-	

					Recessive	1.534 (0.946-2.490)	$8.307×10^{-2}$	-	-	
5	PDGFRB	rs4705415	149517235	A	Additive	0.819 (0.680-0.987)	$3.537×10^{-2}$	No_REF[d]	No_REF	$3.636×10^{-2}$
					Dominant	0.725 (0.555-0.946)	$1.791×10^{-2}$	REF[c]	REF	
					Recessive	0.865 (0.604-1.240)	$4.305×10^{-1}$	-	-	
		rs17708574	149521238	A	Additive	0.822 (0.665-1.016)	$6.967×10^{-2}$	-	-	
					Dominant	0.745 (0.579-0.959)	$2.204×10^{-2}$	rs4705415	$1.997×10^{-1}$	$7.044×10^{-2}$
					Recessive	1.126 (0.594-2.131)	$7.166×10^{-1}$	-	-	
		rs740750	149515074	A	Additive	0.843 (0.704-1.010)	$6.420×10^{-2}$	-	-	$6.747×10^{-2}$
					Dominant	0.737 (0.567-0.959)	$2.310×10^{-2}$	rs4705415	$8.881×10^{-1}$	
					Recessive	0.922 (0.653-1.301)	$6.434×10^{-1}$	-	-	
6	FYN	rs9487729	112151975	A	Additive	0.789 (0.652-0.954)	$1.456×10^{-2}$	No_REF[d]	No_REF	$1.557×10^{-2}$
					Dominant	0.772 (0.594-1.001)	$5.128×10^{-2}$	-	-	
					Recessive	0.655 (0.443-0.966)	$3.303×10^{-2}$	No_REF[d]	No_REF	
7	EGFR	rs6593210	55254186	A	Additive	0.681 (0.484-0.958)	$2.727×10^{-2}$	rs1050171	$6.038×10^{-1}$	$2.516×10^{-2}$
					Dominant	0.669 (0.471-0.951)	$2.497×10^{-2}$	rs1050171	$6.315×10^{-1}$	
					Recessive	0.764 (0.069-8.463)	$8.263×10^{-1}$	-	-	
		rs2740761	55255986	T	Additive	0.682 (0.485-0.960)	$2.807×10^{-2}$	rs1050171	$6.023×10^{-1}$	$2.597×10^{-2}$

					Dominant	0.670 (0.472-0.953)	2.574×10^{-2}	rs1050171	6.300×10^{-1}	
					Recessive	0.763 (0.069-8.454)	8.256×10^{-1}	-	-	
		rs845561	55252708	T	Additive	0.803 (0.647-0.999)	4.841×10^{-2}	rs1050171	6.417×10^{-1}	5.412×10^{-2}
					Dominant	0.802 (0.621-1.036)	9.062×10^{-2}	-	-	
					Recessive	0.620 (0.337-1.140)	1.242×10^{-1}	-	-	
		rs2041647	55183115	A	Additive	1.123 (0.926-1.362)	2.400×10^{-1}	-	-	2.346×10^{-1}
					Dominant	1.031 (0.802-1.324)	8.128×10^{-1}	-	-	
					Recessive	1.678 (1.048-2.687)	3.101×10^{-2}	rs714180	3.101×10^{-2}	
7	MET	rs714180	116319002	A	Additive	1.121 (0.944-1.330)	1.923×10^{-1}	-	-	1.676×10^{-1}
					Dominant	0.988 (0.761-1.282)	9.276×10^{-1}	-	-	
					Recessive	1.500 (1.082-2.079)	1.488×10^{-2}	REF[c]	REF	
11	PDGF-D	rs11226159	103978540	G	Additive	1.332 (1.044-1.700)	2.112×10^{-2}	rs603781	2.274×10^{-1}	2.462×10^{-2}
					Dominant	1.387 (1.051-1.830)	2.088×10^{-2}	rs603781	1.999×10^{-1}	
					Recessive	1.474 (0.672-3.233)	3.332×10^{-1}	-	-	
		rs488753	103976293	C	Additive	1.290 (1.024-1.626)	3.041×10^{-2}	rs603781	2.979×10^{-1}	3.848×10^{-2}
					Dominant	1.355 (1.036-1.771)	2.638×10^{-2}	rs603781	2.365×10^{-1}	
					Recessive	1.333 (0.673-2.638)	4.098×10^{-1}	-	-	

		rs361283	103960620	T	Additive	1.271 (1.020-1.584)	3.271×10^{-2}	rs603781	4.615×10^{-1}	4.008×10^{-2}
					Dominant	1.265 (0.979-1.635)	7.223×10^{-2}	-	-	
					Recessive	1.837 (0.919-3.670)	8.517×10^{-2}	-	-	
		rs10895578	104000193	A	Additive	1.297 (1.013-1.661)	3.888×10^{-2}	rs603781	3.259×10^{-1}	4.412×10^{-2}
					Dominant	1.334 (1.010-1.762)	4.220×10^{-2}	rs603781	2.995×10^{-1}	
					Recessive	1.492 (0.647-3.440)	3.475×10^{-1}	-	-	
		rs12363710	103877311	G	Additive	1.461 (1.000-2.133)	4.977×10^{-2}	rs603781	1.059×10^{-1}	5.967×10^{-2}
					Dominant	1.448 (0.988-2.124)	5.784×10^{-2}	-	-	
					Recessive	NA	9.991×10^{-1}	-	-	
		rs1032233	103838023	A	Additive	0.790 (0.549-1.137)	2.044×10^{-1}	-	-	1.934×10^{-1}
					Dominant	0.863 (0.578-1.289)	4.722×10^{-1}	-	-	
					Recessive	0.144 (0.028-0.747)	2.107×10^{-2}	No_REF[d]	No_REF	
11	BAD	rs11231741	64046885	T	Additive	0.723 (0.549-0.952)	2.096×10^{-2}	rs603781	2.738×10^{-2}	2.117×10^{-2}
					Dominant	0.669 (0.491-0.912)	1.104×10^{-2}	rs603781	1.945×10^{-2}	
					Recessive	0.922 (0.326-2.607)	8.781×10^{-1}	-	-	
12	CCND2	rs10849028	4405850	G	Additive	1.516 (1.082-2.124)	1.561×10^{-2}	rs3217892	5.946×10^{-1}	1.740×10^{-2}
					Dominant	1.524 (1.037-2.241)	3.208×10^{-2}	rs3217892	6.626×10^{-1}	

					Recessive	2.939 (0.867-9.963)	8.354×10^{-2}	-	-	
		rs3217867	4399917	G	Additive	0.659 (0.467-0.931)	1.796×10^{-2}	rs3217892	3.364×10^{-2}	1.602×10^{-2}
					Dominant	0.681 (0.478-0.970)	3.313×10^{-2}	rs3217892	5.500×10^{-2}	
					Recessive	NA	9.990×10^{-1}	-	-	
		rs3217916	4408673	C	Additive	0.833 (0.696-0.996)	4.514×10^{-2}	rs3217892	4.767×10^{-1}	4.964×10^{-2}
					Dominant	0.781 (0.598-1.021)	7.053×10^{-2}	-	-	
					Recessive	0.796 (0.578-1.095)	1.608×10^{-1}	-	-	
		rs3217863	4399496	T	Additive	1.113 (0.922-1.345)	2.643×10^{-1}	-	-	2.886×10^{-1}
					Dominant	1.022 (0.798-1.309)	8.609×10^{-1}	-	-	
					Recessive	1.617 (1.034-2.531)	3.525×10^{-2}	rs3217907	2.313×10^{-1}	
13	FLT1	rs7330109	29020256	T	Additive	0.824 (0.658-1.031)	9.004×10^{-2}	-	-	8.595×10^{-2}
					Dominant	0.751 (0.578-0.976)	3.204×10^{-2}	REF[c]	REF	
					Recessive	1.191 (0.578-2.454)	6.356×10^{-1}	-	-	
		rs11149523	28995630	A	Additive	0.828 (0.663-1.034)	9.554×10^{-2}	-	-	9.484×10^{-2}
					Dominant	0.762 (0.589-0.986)	3.840×10^{-2}	rs7330109	5.560×10^{-1}	
					Recessive	1.162 (0.564-2.394)	6.834×10^{-1}	-	-	
15	IGF1R	rs4966036	99429749	C	Additive	0.879 (0.733-1.055)	1.667×10^{-1}	-	-	1.771×10^{-1}

			Dominant	0.746 (0.568-0.981)	3.598×10^{-2}	REF[c]	REF	
			Recessive	1.020 (0.726-1.432)	9.093×10^{-1}	-	-	
rs2670504	99401590	A	Additive	0.871 (0.730-1.038)	1.231×10^{-1}	-	-	1.343×10^{-1}
			Dominant	0.761 (0.580-0.997)	4.735×10^{-2}	rs4966036	7.025×10^{-1}	
			Recessive	0.940 (0.686-1.288)	7.021×10^{-1}	-	-	
rs8030777	99403952	C	Additive	1.225 (0.982-1.529)	7.219×10^{-2}	-	-	7.700×10^{-2}
			Dominant	1.162 (0.896-1.507)	2.582×10^{-1}	-	-	
			Recessive	2.437 (1.143-5.195)	2.114×10^{-2}	rs11247380	1.873×10^{-1}	
rs1521481	99414423	A	Additive	1.191 (0.953-1.489)	1.253×10^{-1}	-	-	1.342×10^{-1}
			Dominant	1.124 (0.864-1.462)	3.846×10^{-1}	-	-	
			Recessive	2.331 (1.091-4.980)	2.887×10^{-2}	rs11247380	3.001×10^{-1}	
rs2684811	99453563	G	Additive	1.082 (0.902-1.300)	3.958×10^{-1}	-	-	4.451×10^{-1}
			Dominant	0.954 (0.738-1.233)	7.179×10^{-1}	-	-	
			Recessive	1.511 (1.032-2.210)	3.372×10^{-2}	rs11247380	8.297×10^{-1}	
rs7183706	99275189	G	Additive	0.965 (0.783-1.188)	7.342×10^{-1}	-	-	7.487×10^{-1}
			Dominant	1.073 (0.830-1.387)	5.901×10^{-1}	-	-	
			Recessive	0.584 (0.350-0.975)	3.955×10^{-2}	rs11247380	4.288×10^{-2}	

18	BCL2	rs1564483	60794654	T	Additive	0.820 (0.684-0.984)	3.326×10^{-2}	No_REF[d]	No_REF	3.061×10^{-2}
					Dominant	0.779 (0.606-1.002)	5.222×10^{-2}	-	-	
					Recessive	0.763 (0.529-1.102)	1.491×10^{-1}	-	-	
		rs9955190	60878883	G	Additive	1.147 (0.932-1.413)	1.957×10^{-1}	-	-	2.218×10^{-1}
					Dominant	1.338 (1.040-1.720)	2.326×10^{-2}	REF[c]	REF	
					Recessive	0.668 (0.410-1.120)	1.292×10^{-1}	-	-	
		rs2062011	60876114	A	Additive	1.162 (0.919-1.469)	2.109×10^{-1}	-	-	2.397×10^{-1}
					Dominant	1.394 (1.043-1.861)	2.458×10^{-2}	rs9955190	4.066×10^{-1}	
					Recessive	0.682 (0.398-1.167)	1.625×10^{-1}	-	-	
		rs12970840	60915873	T	Additive	1.137 (0.885-1.460)	3.165×10^{-1}	-	-	3.327×10^{-1}
					Dominant	1.318 (0.994-1.747)	5.477×10^{-2}	-	-	
					Recessive	0.384 (0.179-0.826)	1.434×10^{-2}	rs7243091	6.037×10^{-1}	
		rs17841946	60875442	T	Additive	0.861 (0.630-1.176)	3.477×10^{-1}	-	-	3.761×10^{-1}
					Dominant	0.921 (0.653-1.300)	6.393×10^{-1}	-	-	
					Recessive	0.293 (0.089-0.968)	4.407×10^{-2}	rs7243091	3.911×10^{-2}	
22	PDGFB	rs5757573	39633622	C	Additive	0.677 (0.494-0.929)	1.564×10^{-2}	No_REF[d]	No_REF	1.637×10^{-2}
					Dominant	0.686 (0.493-0.955)	2.564×10^{-2}	No_REF[d]	No_REF	

	Recessive	0.247 (0.041-1.485)	1.264×10^{-1}	-	-

Supplement Table 1. The association between aggressive prostate cancer and the loci of the RTK/ERK pathway assessed by the additive, dominant and recessive models, respectively.

a. The P values are based on logistic regression analysis and adjusted for age

b. The conditional P values are based on logistic regression analysis and adjusted for age and reference SNPs

c. The reference SNPs were applied to be adjusted in the conditional analysis

d. Only one SNP with the $P < 0.05$ was shown in additive model (for chromosome 3, 5, 6, 12, 18 and 22), dominant model (chromosome 3 and 22) and recessive model (chromosome 6 and 11)

e. P for Cochran–Armitage trend test

SNP	Genotype	Gleason Score		Aggressive		P	No- Aggressive		P
		Aggressive	No- Aggressive	T (I, II)	T (III, IV)		T (I, II)	T (III, IV)	
rs3217869	GG+GA	7.76±1.09	6.38±0.69	61 (17.38%)	86 (17.48%)		76 (21.90%)	0 (0.00%)	
	AA	7.75±1.15	6.23±0.91	274 (78.06%)	390 (79.27%)	>0.050[a]	249 (71.76%)	0 (0.00%)	NA[a]
	NA			16 (4.56%)	16 (3.25%)	0.612[b]	22 (6.34%)	0 (0.00%)	NA[b]
Undefined				113			0		
Total				956			347		
P[c]		0.897[c]	0.191[c]						
rs11923427	GG+GC	7.78±1.31	6.11±0.58	9 (2.56%)	15 (3.05%)		18 (5.19%)	0 (0.00%)	
	CC	7.55±1.63	6.28±0.86	340 (96.87%)	471 (95.73%)	0.683[a]	327 (94.24%)	0 (0.00%)	NA[a]
	NA			2 (0.57%)	6 (1.22%)	0.629[b]	2 (0.58%)	0 (0.00%)	NA[b]
Undefined				113			0		
Total				956			347		
P[c]		0.467[c]	0.427[c]						
rs6828477	CC+CT	7.62±1.59	6.22±0.82	128 (36.47%)	186 (37.80%)		152 (43.80%)	0 (0.00%)	
	TT	7.51±1.66	6.31±0.88	223 (63.53%)	306 (62.20%)	0.718[a]	195 (56.20%)	0 (0.00%)	NA[a]
	NA			0 (0.00%)	0 (0.00%)	0.718[b]	0 (0.00%)	0 (0.00%)	NA[b]
Undefined				113			0		
Total				956			347		
P[c]		0.303[c]	0.362[c]						
rs17035367	GG+GA	7.52±1.53	6.21±0.77	44 (12.54%)	66 (13.41%)		63 (18.16%)	0 (0.00%)	
	AA	7.56±1.65	6.29±0.87	307 (87.46%)	426 (86.59%)	0.756[a]	284 (81.84%)	0 (0.00%)	NA[a]
	NA			0 (0.00%)	0 (0.00%)	0.756[b]	0 (0.00%)	0 (0.00%)	NA[b]
Undefined				113			0		
Total				956			347		
P[c]		0.803[c]	0.507[c]						
rs12643184	TT+TC	7.72±1.45	6.23±0.79	68 (19.37%)	108 (21.95%)		56 (16.14%)	0 (0.00%)	
	CC	7.43±1.71	6.26±0.88	201 (57.27%)	268 (54.47%)	0.370[a]	220 (63.40%)	0 (0.00%)	NA[a]
	NA			82 (23.36%)	116 (23.58%)	0.620[b]	71 (20.46%)	0 (0.00%)	NA[b]
Undefined				113			0		
Total				956			347		
P[c]		**0.034**[c]	0.808[c]						
rs4705415	AA+AG	7.50±1.67	6.31±0.86	208 (59.26%)	282 (57.32%)		225 (64.84%)	0 (0.00%)	
	GG	7.59±1.63	6.18±0.86	131 (37.32%)	188 (38.21%)	0.716[a]	106 (30.55%)	0 (0.00%)	NA[a]
	NA			12 (3.42%)	22 (4.47%)	0.692[b]	16 (4.61%)	0 (0.00%)	NA[b]
Undefined				113			0		
Total				956			347		
P[c]		0.428[c]	0.193[c]						
rs9487729	AA+AT	7.59±1.64	6.22±0.88	159 (45.30%)	229 (46.54%)		179 (51.59%)	0 (0.00%)	
	TT	7.49±1.69	6.39±0.68	165 (47.01%)	219 (44.52%)	0.610[a]	133 (38.33%)	0 (0.00%)	NA[a]
	NA			27 (7.69%)	44 (8.94%)	0.709[b]	35 (10.09%)	0 (0.00%)	NA[b]
Undefined				113			0		
Total				956			347		

P[c]		0.342[c]	0.060[c]								
rs1050171	AA+AG	7.53±1.77	6.26±0.95	90 (25.64%)		136 (27.64%)		119 (34.29%)		0 (0.00%)	
	GG	7.57±1.57	6.27±0.80	255 (72.65%)		351 (71.34%)	0.580[a]	222 (63.98%)		0 (0.00%)	NA[a]
	NA			6 (1.71%)		5 (1.02%)	0.553[b]	6 (1.73%)		0 (0.00%)	NA[b]
Undefined					113				0		
Total					956				347		
P[c]		0.731[c]	0.920[c]								
rs11231741	TT+TC	7.55±1.80	6.11±1.12	60 (17.09%)		69 (14.02%)		75 (21.61%)		0 (0.00%)	
	CC	7.55±1.60	6.32±0.76	291 (82.91%)		423 (85.98%)	0.244[a]	272 (78.39%)		0 (0.00%)	NA[a]
	NA			0 (0.00%)		0 (0.00%)	0.244[b]	0 (0.00%)		0 (0.00%)	NA[b]
Undefined					113				0		
Total					956				347		
P[c]		0.999[c]	0.059[c]								
rs603781	TT+TC	7.46±1.69	6.29±0.88	182 (51.85%)		267 (54.27%)		150 (43.23%)		0 (0.00%)	
	CC	7.65±1.56	6.26±0.83	169 (48.15%)		225 (45.73%)	0.529[a]	197 (56.77%)		0 (0.00%)	NA[a]
	NA			0 (0.00%)		0 (0.00%)	0.529[b]	0 (0.00%)		0 (0.00%)	NA[b]
Undefined					113				0		
Total					956				347		
P[c]		0.073[c]	0.764[c]								
rs3217892	AA+AG	7.42±1.77	6.33±0.90	104 (29.63%)		139 (28.25%)		73 (21.04%)		0 (0.00%)	
	GG	7.60±1.55	6.24±0.82	193 (54.99%)		274 (55.69%)	0.749[a]	216 (62.25%)		0 (0.00%)	NA[a]
	NA			54 (15.38%)		79 (16.06%)	0.907[b]	58 (16.71%)		0 (0.00%)	NA[b]
Undefined					113				0		
Total					956				347		
P[c]		0.158[c]	0.441[c]								
rs5757573	CC+CT	7.43±1.64	6.38±0.77	46 (13.11%)		65 (13.21%)		63 (18.16%)		0 (0.00%)	
	TT	7.57±1.63	6.25±0.87	305 (86.89%)		427 (86.79%)	>0.05[a]	284 (81.84%)		0 (0.00%)	NA[a]
	NA			0 (0.00%)		0 (0.00%)	>0.05[b]	0 (0.00%)		0 (0.00%)	NA[b]
Undefined					113				0		
Total					956				347		
P[c]		0.385[c]	0.257[c]								

Supplementary Table 2. The association between 12 identified loci and aggressive prostate cancer risk on the basis of Gleason Score and clinical stage

a. The P value for two groups (GG+GA and AA) for clinical stage
b. The P value for three groups (GG+GA, AA and NA) for clinical stage
c. P value for the group of GG+GA and AA for Gleason Score

			Linear model			KWH test
**SNP**	**Gene**	**Statistic**	**P-value**	**FDR**	**beta**	**P-value**
rs3217869	**CCND2**	-3.4618	**0.00075**	0.0105	-0.2953	**0.00879**
rs3217869	RAD51AP1	2.25616	0.02592	0.18143	0.05796	0.155
rs3217869	C12orf5	1.70871	0.09016	0.34652	0.05121	0.23
rs3217869	AKAP3	1.66292	0.09901	0.34652	0.02234	0.0922
rs3217869	DYRK4	1.04467	0.29833	0.77279	0.03423	0.111
rs3217869	PARP11	0.91995	0.35949	0.77279	0.04553	0.345
rs3217869	C12orf4	0.86043	0.39131	0.77279	0.02763	0.568
rs3217869	FGF6	0.66611	0.50665	0.77279	0.00774	0.27
rs3217869	FGF23	0.58241	0.56141	0.77279	0.00722	0.449
rs3217869	KCNA1	-0.5155	0.60719	0.77279	-0.0059	0.634
rs3217869	GALNT8	0.14609	0.8841	0.96695	0.00208	0.605
rs3217869	PRMT8	-0.0511	0.9593	0.96695	-0.0006	0.675
rs3217869	KCNA6	-0.0415	0.96695	0.96695	-0.0007	0.574
rs3217869	KCNA5	-0.7298	0.46697	0.77279	-0.0207	0.138

Supplementary Table 3. Statistical analysis of the association between SNP rs3217869 and the genes located within 2 Mb of rs3217869. KWH test, Kruskal–Wallis H test.
